# Signature of dislocations and stacking faults of face-centred cubic nanocrystals in coherent X-ray diffraction patterns: a numerical study^1^

**DOI:** 10.1107/S1600576715005324

**Published:** 2015-04-16

**Authors:** Maxime Dupraz, Guillaume Beutier, David Rodney, Dan Mordehai, Marc Verdier

**Affiliations:** a Université Grenoble Alpes, SIMAP, Grenoble, F-38000, France; b CNRS, SIMAP, Grenoble, F-38000, France; c Institut Lumière Matière, Université Lyon 1, CNRS, UMR 5306, Villeurbanne, F-69622, France; d Department of Materials Engineering, Technion – Israel Institute of Technology, Haifa, 32000, Israel

**Keywords:** dislocations, stacking faults, face-centred cubic nanocrystals, coherent X-ray diffraction

## Abstract

Crystal defects induce strong distortions in diffraction patterns. A single defect alone can yield strong and fine features that are observed in high-resolution diffraction experiments such as coherent X-ray diffraction. The case of face-centred cubic nanocrystals is studied numerically and the signatures of typical defects close to Bragg positions are identified. Crystals of a few tens of nanometres are modelled with realistic atomic potentials and ‘relaxed’ after introduction of well defined defects such as pure screw or edge dislocations, or Frank or prismatic loops. Diffraction patterns calculated in the kinematic approximation reveal various signatures of the defects depending on the Miller indices. They are strongly modified by the dissociation of the dislocations. Selection rules on the Miller indices are provided, to observe the maximum effect of given crystal defects in the initial and relaxed configurations. The effect of several physical and geometrical parameters such as stacking fault energy, crystal shape and defect position are discussed. The method is illustrated on a complex structure resulting from the simulated nanoindentation of a gold nanocrystal.

## Abstract 2

Crystal defects can be identified by their fingerprint in coherent X-ray diffraction patterns. Realistic defects in face-centred cubic nanocrystals are studied numerically, revealing various signatures in diffraction patterns depending on the Miller indices and providing an identification method.

## 1. Introduction   

The microstructure of materials plays a large role in determining their physical properties (Hull & Bacon, 2001 ▶; Hirth & Lothe, 1968 ▶). Even in a small crystallite, elastic strain and crystal defects are of primary importance, in particular in small-scale structures. For instance, electron transport properties and superconductivity (Ying *et al.*, 2013 ▶) are strongly affected by dislocations, and the mechanical response of crystals is driven by dislocation motion, such that the presence of a few dislocations and their nature strongly impact the mechanical properties of submicrometre crystals (Bei *et al.*, 2008 ▶). Tailoring and monitoring the microstructure of materials is therefore of primary importance in order to guarantee the best performance of nanodevices.

A variety of experimental techniques are available for evidencing and identifying crystal defects. Among them, transmission electron microscopy (TEM) is routinely used to produce various imaging contrasts of dislocations in real space by selecting pertinent diffraction vectors, according to well known invisibility criteria (Wiliams & Carter, 1996 ▶). It has atomic resolution and thus can evidence individual crystal defects. However, the use of TEM is hindered by strong experimental constraints on the sample environment and thickness. These restrictions are relaxed for X-rays, which thus have a great potential for the study of defects in crystals.

Elastic diffuse scattering of X-rays (Krivoglaz, 1969 ▶), neutrons (Moisy-Maurice *et al.*, 1981 ▶) or electrons (Zhou *et al.*, 2005 ▶) has been used since the 1970s to study crystals containing defects with displacement fields. Near Bragg positions (Huang diffuse scattering), it provides valuable information on long-range lattice distortions, far away from defects. Further away from Bragg peaks, asymptotic diffuse scattering (also known as Stokes–Wilson scattering) can in some cases provide information on shorter-range lattice distortions (Dederichs, 1971 ▶). However, the signature of defect cores, so-called Laue scattering (Larson & Schmatz, 1980 ▶) or structural diffuse scattering (Ehrhart *et al.*, 1982 ▶), whose extent is limited in real space, is very diffuse in reciprocal space (Krivoglaz, 1969 ▶; Fultz & Howe, 2007 ▶) and orders of magnitude weaker than the Huang diffuse scattering. Despite this limitation, it has been used successfully on a large number of systems. In the early 1970s X-ray scattering from single and clusters of point defects was investigated theoretically (Dederichs, 1973 ▶; Trinkaus, 1972 ▶). A few years later, Huang diffuse scattering from dislocation loops was considered, both experimentally (Larson & Schmatz, 1980 ▶; Larson & Young, 1987 ▶) and numerically (Ehrhart *et al.*, 1982 ▶). More recently, the calculated and measured X-ray diffuse scattering from threading dislocations in epitaxial GaN layers provided a precise estimation of the dislocation density and the relative proportion of dislocations (edge or screw type), in good agreement with already existing destructive methods (Barchuk *et al.*, 2010 ▶). Since neutrons and X-rays probe large volumes of materials containing many defects of various types, the interpretation of diffuse scattering usually assumes a model for the dominant defects and a rather large density of them. In the case of dislocation loops or stacking faults, diffuse scattering has to be averaged over all possible loop orientations. Interpreting correctly the shape and symmetry of the elastic diffuse scattering requires the use of single crystals and careful averaging procedures. The smaller probe size (∼50 nm) achievable with electron beams has allowed the measurement of electron diffuse scattering from single defects and individual dislocation loops (Kirk *et al.*, 2005 ▶, 2006 ▶). Similar studies with X-rays are now being developed thanks to the progress of X-ray focusing optics.

In the past decade, the availability of intense coherent X-ray beams from third-generation synchrotron facilities has allowed the emergence of a very attractive technique to probe the microstructure of crystals: coherent X-ray diffraction (CXD) (Livet, 2007 ▶; Sutton, 2008 ▶). In Bragg geometry, it probes the deviation from the perfect crystal lattice and has been successfully used to characterize elastic strain in isolated crystals (Beutier *et al.*, 2012 ▶) and to show the presence of crystal defects such as stacking faults (Chamard *et al.*, 2008 ▶; Favre-Nicolin *et al.*, 2010 ▶) and dislocation loops (Jacques *et al.*, 2011 ▶). Recently, the same principles have been applied to electrons, and the first measurements of coherent electron diffraction have been reported (Huang *et al.*, 2008 ▶).

Following Sayre’s principle (Sayre, 1952 ▶), CXD has been turned into an imaging technique known as coherent diffraction imaging (CDI) (Miao *et al.*, 1999 ▶): by oversampling the diffraction pattern and with the help of iterative phase retrieval algorithms, the scattering function that encodes the crystal density and, in the Bragg case, a projection of the displacement field (Robinson & Harder, 2009 ▶; Pfeifer *et al.*, 2006 ▶) can be recovered. In the latter case, the three-dimensional measurement of the reciprocal space in the vicinity of a Bragg reflection yields a three-dimensional image of the strained crystal (Pfeifer *et al.*, 2006 ▶) with a typical resolution of a few nanometres and a strain sensitivity better than 10^−3^ (Watari *et al.*, 2011 ▶). Several Bragg reflections can be combined to recover all the components of the displacement field (Newton *et al.*, 2010 ▶). While this method of characterization is now well established for weakly strained systems, its application to highly strained systems has so far been successful only for a limited number of cases owing to the strong inhomogeneity of the phase to be recovered (Minkevich *et al.*, 2008 ▶; Diaz *et al.*, 2010 ▶; Vaxelaire *et al.*, 2010 ▶). In its original version, CDI was restricted to finite objects, because phase retrieval algorithms need a real-space constraint (such as a finite support constraint) in order to converge. In recent years, this limitation has been lifted by the introduction of ptychography, a scanning version of CDI: with scanning steps smaller than the beam size, sufficient redundancy is obtained in the data to allow the reconstruction of extended objects with the help of dedicated algorithms (Rodenburg & Faulkner, 2004 ▶). In Bragg conditions it has been used to reconstruct the strain field of extended objects (Hruszkewycz *et al.*, 2012 ▶; Godard *et al.*, 2011 ▶) and to reconstruct a single dislocation and its associated strain field (Takahashi *et al.*, 2013 ▶); however, the case of multiple defects is still out of reach.

CDI and ptychography often fail to provide a real-space reconstruction quickly, while a rapid evaluation of data might be needed during experiments. This is particularly true in the case of Bragg ptychography, which requires a considerable quantity of data. Moreover, for both CDI and ptychography the definition of a good input for the initialization of the inversion cycles is of primary importance. There is thus an interest in understanding diffraction patterns qualitatively and interpreting them directly in reciprocal space. In particular, during *in situ* mechanical loading of a sample (Beutier *et al.*, 2013 ▶; Ren *et al.*, 2014 ▶), one would like to witness the first plastic events by measuring a CXD pattern and interpreting it on the fly. Here we use this direct approach, which consists in first modelling the object in real space and second computing the corresponding reciprocal-space pattern, and try to identify characteristic signatures of defects that can be observed in experimental CXD data. While obtaining the displacement field of the sample in real space provides a more comprehensive picture, all the information is present in reciprocal space, and it should in principle be possible to extract valuable information on the nature of defects within the sample without the difficulty of reverting to real space.

So far only a few studies have been carried out on individual defects with CXD: misfit dislocations in an epitaxial SiGe thin film (Robinson *et al.*, 2005 ▶), Frank dislocation loops in silicon (Jacques *et al.*, 2011 ▶), a single dislocation in silicon (Takahashi *et al.*, 2013 ▶), stacking faults in semiconductor nanowires (Chamard *et al.*, 2008 ▶; Favre-Nicolin *et al.*, 2010 ▶), and dislocations in charge and spin density waves (Le Bolloc’h *et al.*, 2005 ▶; Jacques *et al.*, 2009 ▶). In the present paper, focused on common face-centred cubic (f.c.c.) metals, we demonstrate that CXD can be used to identify single defects directly from their signature in the diffraction pattern, provided the Bragg reflection is well chosen. We establish that, similarly to TEM (Wiliams & Carter, 1996 ▶), the careful choice of diffraction conditions is essential when it comes to highlighting specific defects.

We consider first the cases of single defects: a single defect can induce strong modifications of the diffraction pattern and therefore a good understanding of these elementary cases is necessary before investigating crystals with multiple defects. There are a large variety of crystal defects. We focus here on the most common ones for f.c.c. crystals. After introducing the tools and methods used for this study in §2, we start with the screw and edge dislocations (§§3.1 and 3.2 respectively), then the stacking faults (§3.3), and finally the Frank and prismatic dislocation loops (§§3.4 and 3.5, respectively), crystalline defects commonly introduced in metals by irradiation (Stoller *et al.*, 1992 ▶), rapid thermal treatments (quench) or mechanical loading (indentation). In §3.6 we investigate the effect of the size and shape of the crystal, and in §3.7 we discuss the effect of the position of the defect in the crystal. Finally, we apply our methodology in §4 to the analysis of a more complex structure resulting from the simulated nanoindentation of a gold nanocrystal.

## 2. Tools and methods   

A common method to analyse CXD measurements is to model the diffracting object with the finite element method (FEM) and to calculate the CXD pattern by Fourier transforming a modified electronic density (Diaz *et al.*, 2010 ▶; Beutier *et al.*, 2012 ▶). FEM uses a continuous description of matter and thus has the advantage of allowing the modelling of large crystals. However, this continuous description is not able to deal with plasticity, despite a possible correction of the elastic strain by taking into account the plastic relaxation (Proudhon *et al.*, 2010 ▶). It is therefore not well suited to the study of faulted crystals. Alternatively, analytical models have been used to explain the effect of ‘perfect’ crystal defects in CXD patterns. While such a simple model gives a reasonable description of defects in electronic crystals (Le Bolloc’h *et al.*, 2005 ▶; Jacques *et al.*, 2009 ▶), it does not take into account the dissociation of dislocations into partials, which can have a strong effect on the CXD patterns. In this study we use an atomistic description of matter, in order to accurately model crystal defects. This comes at the price of the size of the studied objects, but progress in atomic scale modelling and X-ray focusing optics has allowed a convergence of the scales of individual objects that these techniques can study (Schroer *et al.*, 2008 ▶). With an electron beam it is possible to deal with even smaller scales, and using coherent electron diffraction beams Huang *et al.* (2008 ▶) were able to extract valuable information on the surface relaxation of gold nanocrystals of less than 5 nm in diameter. Here we deal with crystals of typical size of the order of a few tens of nanometres.

**Figure 1:**
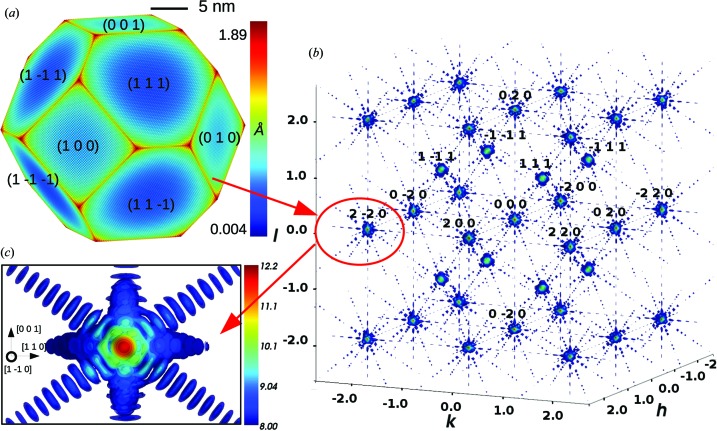
(*a*) Defect-free gold nanocrystal of Wulff geometry and size 30 × 30 × 30 nm. The colour scale encodes the magnitude of displacements of the surface atoms after relaxation. (*b*) Three-dimensional intensity map of the corresponding reciprocal space. (*c*) Zoom on the Bragg reflection **g** = 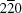. The area of the reciprocal space is kept to the same value in all figures and is equal to 0.045 × 0.0675 Å^−1^.

Molecular statics is used to simulate nanocrystals of common f.c.c. transition metals (aluminium, copper, silver, gold and nickel), modelled with embedded atom method (EAM) potentials (Mishin *et al.*, 1999 ▶, 2001 ▶; Williams *et al.*, 2006 ▶; Grochola *et al.*, 2005 ▶) that accurately reproduce elastic properties as well as surface and stacking fault energies. The geometry considered here consists of a free-standing equilibrium-shaped crystallite, which minimizes the surface energy through a Wulff construction (Winterbottom, 1967 ▶) (see Fig. 1 ▶ *a*). Owing to the low surface energy of its {111} and {100} facets, this geometry exhibits a remarkable stability and is commonly observed experimentally (Mordehai, Lee *et al.*, 2011 ▶; Sadan & Kaplan, 2006 ▶). Since we want to highlight the effect of defects we do not consider here the case of pre-strained particles, for instance when a crystallite is in an epitaxial relationship with a substrate. The reference crystallite considered throughout this study contains 10^6^ atoms and measures approximately 30 × 30 × 30 nm. The defects are introduced with defined characters: edge or screw dislocations, Frank and prismatic dislocation loops, and stacking faults. The system is relaxed by energy minimization at 0 K using a quenched dynamical algorithm (Rodney *et al.*, 2005 ▶). The large difference between the stacking fault energies (SFEs) of the selected materials is expected to strongly influence the characteristics of the crystalline defects (Rodney *et al.*, 2005 ▶; Groves & Kelly, 1963 ▶; Smallman & Green, 1964 ▶). Understanding the influence of this parameter on relaxation and its corresponding effect on diffraction patterns is one of the goals of the present study. We also focus on the ability of CXD to determine the parameters that define a dislocation, its Burgers vector, line direction, and slip and dissociation planes. The three-dimensional CXD patterns are calculated by summing the amplitudes scattered by each atom with its phase factor, following a kinematic approximation: 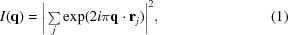
where **q** is the scattering vector and **r**_*j*_ the position of atom *j*. Here we discarded the atomic scattering factor as we are dealing with mono-element materials. The kinematic approximation is justified by the relatively small size of the crystals studied here and the large perturbation of the perfect lattice caused by the defects in such small volumes. Equation (1) assumes a plane wave illumination, which is a reasonable approximation for most experimental conditions on such small objects at synchrotron radiation facilities, even with microfocusing optics (Mastropietro *et al.*, 2011 ▶). Equation (1) also assumes fully coherent scattering. Absorption and refraction effects are not considered in this study.

For objects of size *L* and lattice parameter *a*, the reciprocal space must be probed with a step no larger than *a*/2*L* in reciprocal lattice units (r.l.u.) in order to resolve the smallest possible features in reciprocal space. In the case of 30 nm crystals of common f.c.c. transition metals, *a*/2*L* ≃ 0.02 r.l.u. (0.006 Å^−1^ in the case of a 30 nm copper nanocrystal), but we typically sample the diffraction pattern with a step size of 0.0015 r.l.u. (0.00045 Å^−1^) to obtain smoother representations. Given the large number of atoms (∼10^6^) and the similarly large number of points in reciprocal space for which the calculation is performed (typically 100 × 100 × 100 = 10^6^ for each pattern), the computation is performed with a graphical processing unit (GPU), which allows massive parallelism. Current GPUs that include up to 2500 cores are particularly efficient for computing large diffraction maps. Equation (1) was computed with the *PyNX* code (Favre-Nicolin *et al.*, 2011 ▶) on an NVidia GTX 580 GPU, which achieves a speed of calculation of up to 4 × 10^10^ atoms reflections s^−1^. This is almost three orders of magnitude higher than with a single core central processing unit (CPU). For our usual calculations [sum in equation (1) for 10^6^ atoms and 10^6^ points in reciprocal space], the calculation of the three-dimensional CXD pattern around a Bragg position takes about 25–30 s. Such calculations can easily be performed during experiments to help data evaluation.

In the present study, all the calculations are carried out in the vicinity of Bragg positions **g** defined by their Miller indices *hkl*. **g** is a particular case of the generic scattering vector **q**, and in the following it will be referred to as the diffraction vector. The effect of dislocations on CXD patterns arises from their corresponding atomic displacement field **u**(**r**) with respect to the lattice of the perfect crystal. A commonly reported method in electron microscopy is to use a diffraction vector parallel to the dislocation line (Wiliams & Carter, 1996 ▶). The invisibility condition **g**·**b** = 0 (Wiliams & Carter, 1996 ▶; Head *et al.*, 1967 ▶; Steeds, 1966 ▶), where **b** is the Burgers vector of the dislocation, is also extensively employed in this study, in particular to show the effect of dissociation. According to equation (1), it is clear that crystal defects distort the diffraction pattern when they produce a displacement field that is not perpendicular to the diffraction vector **g**, and conversely one can expect a maximal effect when the main direction of the displacement field is parallel to **g**. However, in most cases, the detailed distortion cannot be predicted easily: already in infinite or semi-infinite isotropic materials the displacement field can have a complex analytical form, and the situation is further complicated by the relaxation of the system, which is affected by the interatomic potentials and the tension-free mechanical equilibrium conditions at the free surfaces. All these considerations explain the need to rely on an atomistic description with reliable interatomic potentials for a more complete and accurate description of the problem.

## 3. Simulations on f.c.c. nanocrystals   

Fig. 1 ▶ illustrates a 30 × 30 × 30 nm perfect (strain and defect-free) copper nanocrystal in Wulff geometry after relaxation (Fig. 1 ▶ *a*) and the corresponding three-dimensional intensity map of its reciprocal space calculated according to equation (1) (Fig. 1 ▶ *b*). In the following it will be referred to as the reference nanocrystal.

It is important to notice that the assumption of a strain-free and defect-free object for the reference nanocrystal is only valid in the initial state, *i.e.* before the nanocrystal has been relaxed by energy minimization. Upon relaxation a contraction of the surface atoms towards the bulk can be observed (Huang *et al.*, 2008 ▶). As illustrated in Fig. 1 ▶(*a*), the motion of the surface atoms is strongly correlated to their coordination number, explaining why such high displacement is observed for corner and edge atoms. Additionally, since the {100} surface atoms are less coordinated than the {111} surface atoms, the {100} facets tend to contract more towards the bulk than the {111} facets. Coherent X-ray diffraction is very sensitive to the atomic structure of the nanocrystal surfaces, and characteristic features due to the contraction of nanocrystal facets during relaxation can be observed on the calculated CXD patterns. They also depend on the *hkl* indices of the Bragg reflection. However, we will see in the next section that the introduction of a single defect within the crystallite produces an even stronger signature on CXD patterns. As a result, in the case of defective nanocrystals, even if the contraction of surface atoms still has some effects on the calculated diffraction patterns, they can be assumed negligible in comparison to the features associated with the defect and its corresponding displacement field. Since we deal only with defective nanocrystals in the next sections, the effect of the displacement of surface atoms and the corresponding surface strain is not further addressed in this work.

If we were dealing with a perfect crystal, the CXD patterns around all allowed Bragg reflections would be identical to the CXD pattern at the origin of reciprocal lattice. Here the surface relaxation is weak enough that the CXD patterns still display essentially the same features, which can be observed for instance around **g** = 2
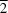
0 (Fig. 1 ▶ *c*). The intensity is maximal at the Bragg position. The diffraction pattern forms streaks along the {111} and {100} directions due to the crystal facets, and these streaks are fringed because of the finite size of the crystal. We call *I*_0_ = *N* ^2^, where *N* is the number of atoms in the nanocrystal, the intensity scattered at the exact Bragg position by the perfect crystal. In the following we will use this reference intensity to quantify the effect of crystal defects. For the reference nanocrystal all the calculations around a given Bragg reflection are performed in a reciprocal space volume of 0.045 × 0.045 × 0.0675 Å^−1^. Since all the calculations presented in §3 are performed on crystals whose size and number of atoms are similar to the reference crystal, the investigated area of reciprocal space in §3 is always the same and equal to 0.045 × 0.0675 Å^−1^ [area within the black rectangle surrounding a CXD pattern, such as Fig. 1 ▶(*c*)]. Consequently, in order to simplify the figures, axes are not shown on the reciprocal space figures. Additionally, the dynamical range of intensities is limited to 4.2 decades, which is typical for a CXD experiment. Similarly, the intensity dynamical range is kept to the same value all through §[Sec sec3]3.

### 3.1. Screw dislocations   

For a screw dislocation, the displacement field **u**(**r**) is parallel to the dislocation line and the Burgers vector **b**, such that **u** is proportional to **b** and **g**·**b** = 0 is an invisibility condition for a perfect screw dislocation. However, this condition is not strictly fulfilled in the vicinity of **g** (**q** ≠ **g**), such that a weak distortion of the Bragg spot cannot be excluded. This distortion could lead to strong diffuse scattering in the case of many defects measured with an incoherent X-ray beam.

**Figure 2:**
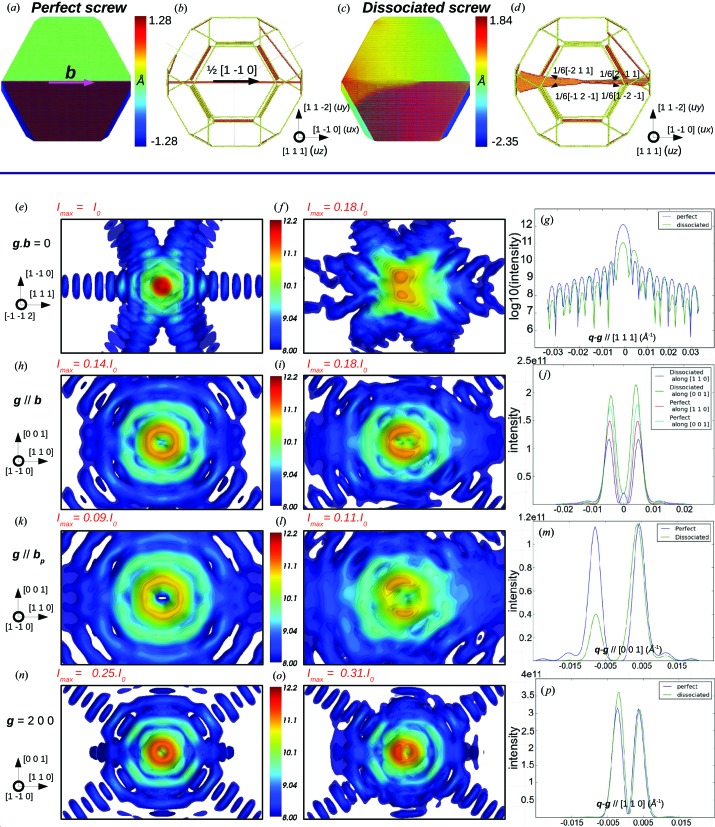
Screw dislocation in a 30 × 30 × 30 nm copper crystal with Wulff geometry. (*a*) and (*c*) The colour scale shows the *u_x_* component of the atomic displacement field for both initial and relaxed configurations. (*b*) and (*d*) Perfect screw dislocation with **b** = 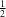
[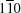] and dissociation of the perfect dislocation in two sets of Shockley partials in the (111) and (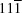) planes. Only the defect, edge and corner atoms are shown. Calculated CXD patterns with **g**·**b** = 0 (**g** = 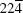) for a perfect (*e*) and dissociated dislocations (*f*). (*g*) Intensity along [111] (log scale). Calculated CXD patterns with **g**||**b** (**g** = 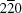) for a perfect (*h*) and dissociated dislocations (*i*). (*j*) Intensity along [001]. Perfect (*k*) and dissociated dislocations (*l*) and intensity along [001] (*m*) with **g**||**b**_p_ (**g** = 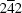). Perfect (*n*) and dissociated dislocations (*o*) and intensity (*p*) along [110] for general **g** (**g** = 200). The area of the reciprocal space is kept to the same value in all figures and is equal to 0.045 × 0.0675 Å^−1^.

The screw dislocation simulated here has a Burgers vector **b** = 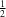
[1
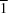
0]. It is introduced at the centre of the nanocrystal with its associated displacement field in an infinite isotropic medium: *u_x_* = **u**||**b** = *b*θ/2π. The initial configuration is relaxed by quenched molecular dynamics simulations to get the relaxed positions and the corresponding atomic displacement field. Figs. 2 ▶(*a*) and 2 ▶(*c*) show the *u_x_* component of the atomic displacement field, *i.e.* parallel to the Burgers vector and line direction, for both the initial and the relaxed configuration: it is exactly equal to ±*b*/2 in the initial configuration, while it increases during the relaxation process, partly because of the dissociation into partial dislocations but also because of the contraction of surface atoms described in the previous section. In Figs. 2 ▶(*b*) and 2 ▶(*d*), atoms are colour coded according to their coordination number and only the defective, corner and edge atoms are shown. The dislocation dissociates in both {111} planes that contain the Burgers vector, *i.e*. the (111) and (11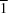) planes, and thus adopts a nonplanar configuration (Fig. 2 ▶ *d*). At the end of the relaxation process two sets of two partial Shockley dislocations (Hull & Bacon, 2001 ▶) are stabilized within the nanocrystal, with respective Burgers vectors of 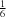
[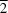
11] and 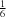
[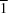
2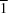] in the (111) plane and 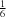
[2
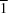
1] and 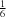
[1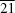] in the (11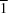) plane. This crossed configuration is more energetically favourable than the configuration with coplanar stacking faults because of the negative energy of the intersecting node (Rasmussen *et al.*, 1997 ▶). The *u_x_* component of the atomic displacement field is exactly equal to *b*/4 within the (111) stacking fault ribbon. The contraction of the surface atoms towards the bulk, which is particularly high for corner and edge atoms owing to their low coordination number, is similar to the case of the defect-free crystal.

The invisibility criterion **g**·**b** = 0 is selected to show the effect of dissociation. With such a diffraction condition, when the dislocation is not dissociated (Fig. 2 ▶ *e*), the Bragg peak is undistorted compared to that of a perfect crystal. This is not the case for the dissociated dislocation, which yields a splitting of the Bragg peak along **b** (Fig. 2 ▶ *f*). For low *h*, *k*, *l* values (typically for *h* + *k* + *l* < 4) no splitting can be seen, but the elongation of the Bragg peak along **b** is clearly visible. This demonstrates that dissociation can be unambiguously shown using CXD. For this particular diffraction vector, interference between the faulted planes and the facets also induces strong distortions in the fringes along the [111] and [11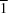] directions. It is well known that stacking faults create streaks along the normal of their plane, but here the effect is modulated by the form factor of the crystal. A closer look at the intensity profile along the [111] direction (Fig. 2 ▶ *g*) reveals that the fringe intensity decreases steadily as we move away from the Bragg position in the case of a perfect dislocation, while the intensity profile is more erratic in the case of a dissociated dislocation, with a drop of intensity every two fringes. The doubling of the fringe periodicity can be explained by the position of the stacking fault at the centre of the crystallite, which implies that the distance between two (111) facets is twice the distance between a (111) facet and the (111) faulted plane. As the extent in reciprocal space is inversely proportional to that in real space, the period of the fringes produced by the stacking fault fringes is therefore twice the period of the fringes induced by the crystal facets.

The case **g**||**b** shown in Figs. 2 ▶(*h*) and 2 ▶(*i*) for a perfect and a dissociated dislocation, respectively, exhibits a very characteristic signature on the CXD pattern: at the Bragg position, the intensity vanishes (completely for the perfect dislocation, almost completely for the dissociated dislocation). Instead we observe a ring-shaped distribution of intensity around the Bragg position. For a perfect screw dislocation at the centre of an isotropic material, the symmetry would impose a uniaxial distribution of intensity with axis parallel to the dislocation line. Here the anisotropy of the elasticity tensor slightly distorts the perfect ring (Fig. 2 ▶ *h*). The ring size is strongly dependent on the Miller indices of the reflection and on the crystal size. For **g** = 2
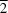
0 and a 30 × 30 × 30 nm crystallite, the ring diameter is *d* = 0.01 Å^−1^. Micro- or nanocrystals observed experimentally are often one order of magnitude larger (Beutier *et al.*, 2012 ▶; Mordehai, Lee *et al.*, 2011 ▶), resulting in a ring diameter ten times smaller in reciprocal space. Our ability to resolve such features experimentally will be discussed in the last section. For a dissociated dislocation (Fig. 2 ▶ *i*), a ring-shaped pattern is still obtained, but the distribution of intensity in the ring is more contrasted and the intensity at the centre does not completely vanish anymore (it is in fact not strictly zero in the case of the perfect dislocation, but it increases by a factor of 25 when the dislocation dissociates). Owing to the dissociation into partials, the strain around the dislocations is inhomogeneous, but one can assume that this inhomogeneity does not produce a sufficient effect to affect the shape of the CXD pattern. However, the effect of dissociation can clearly be seen in the distribution of intensity on the CXD pattern. A tetragonal distribution, typical of the [1
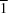
0] zone axis, is observed in both cases, but in the case of the perfect dislocation it looks almost hexagonal, reflecting the crystal shape projected along the dislocation axis, since the latter induces no strong asymmetry. In the dissociated case, the symmetry of the defect structure induces a significant change of distribution and its anisotropy dominates the symmetry of the crystal shape. For the latter, the maxima of intensity are along [001], which is a good indication of the anisotropy of the strain along the [001] and [110] axes. The intensity profile along [001] (Fig. 2 ▶ *j*) reveals an increase of the maxima of intensity of about 20%, while the intensity of the maxima along [110] decreases by 25%.

When **g** is parallel to a partial Burgers vector **b**_p_ (Figs. 2 ▶ *k* and 2 ▶ *l*) the resulting diffraction pattern for a perfect dislocation is very similar to the case **g**||**b**, with a ring-shaped pattern oriented along **b**. After dissociation, a ring-shaped pattern is still observed, but now oriented along the partial Burgers vector **b**_p_. For these particular diffraction conditions, we can infer that the Shockley partial is seen as a single perfect dislocation with a signature independent of the other partial and of the stacking fault.

Finally, for a general **g** (Figs. 2 ▶ *n* and 2 ▶ *o*), a perfect screw dislocation still produces a ring-shaped diffraction pattern with an axis along **b**. A relaxed system yields a distorted and disoriented ring-shaped pattern. Under such diffraction conditions, all four Shockley partials contribute to the diffraction pattern. However, unlike the particular cases detailed above, the ring axis is dependent on **g** but not directed along any particular direction.

The screw dislocation is therefore a relatively simple case to understand. For a perfect dislocation, only two cases are possible. When the extinction condition **g**·**b** = 0 is fulfilled, the dislocation remains invisible and the resulting pattern is similar to that of a perfect crystal. For any other diffraction vector, the characteristic signature of a perfect dislocation is a ring-shaped pattern oriented along **b**. Analysis of CXD patterns produced by a dissociated dislocation is not as straightforward, but it appears very clear that the diffraction conditions where **g** is perpendicular to **b** or parallel to a potential **b**_p_ are best suited to show the effect of dissociation. For diffraction vectors yielding a ring-shaped pattern, the anisotropic distribution of intensity and the increase of the maximum of intensity (by approximately 20%) and of the intensity in Bragg position are also good indicators of a dissociation.

### 3.2. Edge dislocations   

**Figure 3:**
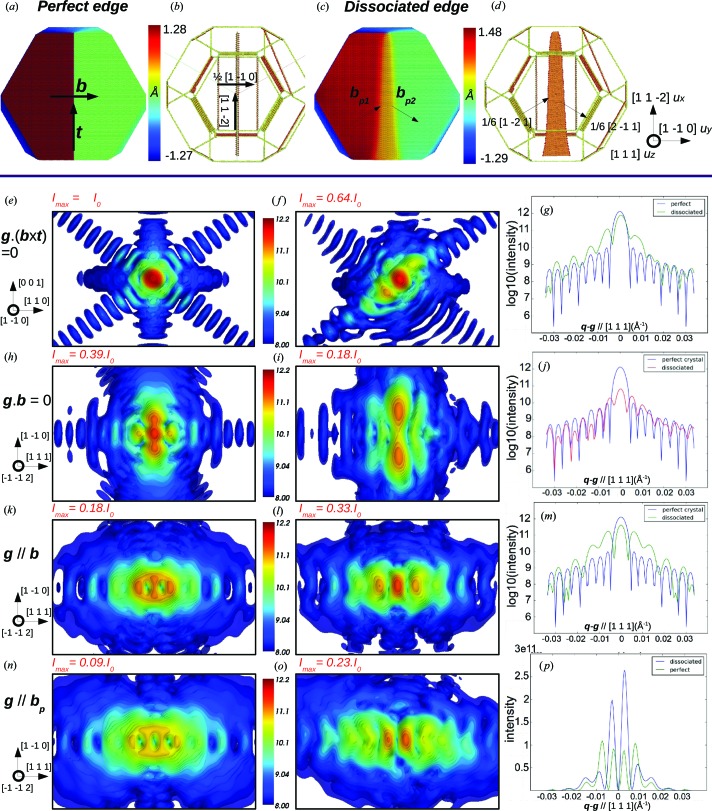
Edge dislocation in a 30 × 30 × 30 nm copper crystal. (*a*) and (*c*) 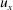 component of the atomic displacement field for both initial and relaxed configurations. (*b*) and (*d*) Perfect edge dislocation with **b** = 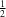
[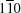] and **t** = [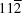] and dissociation of the perfect dislocation into two Shockley partials in the (111) plane. Only the defect, edge and corner atoms are shown. Calculated CXD pattern for a perfect (*e*) and dissociated (*f*) dislocations. (*g*) Intensity along [111] for perfect and dissociated dislocations (log scale) with **g**·**b** = 0 and **g**·(**b** × **t**) = 0 (**g** = 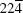). Perfect (*h*) and dissociated dislocations (*i*) and intensity along [111] for both cases (*j*) with **g**·**b** = 0 and **g**·(**b** × **t**) ≠ 0 (**g** = 224). Perfect (*k*) and dissociated dislocations (*l*) and intensity along [111] for a defect-free crystal and dissociated dislocation (log scale) (*m*) with **g**||**b** (**g** = 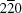). Perfect (*n*) and dissociated dislocations (*o*) and intensity along [111] for both cases (*p*) with **g**·**b**_p_ (**g** = 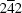). The selected area of the reciprocal space is kept to the same value in all figures and is equal to 0.045 × 0.0675 Å^−1^.

Now we introduce an edge dislocation at the centre of the reference crystal. The Burgers vector is **b** = 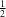
[1
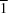
0], which is by definition perpendicular to the dislocation line direction **t** = [11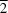]. Similarly to a perfect screw dislocation, an edge dislocation dissociates during relaxation into two Shockley partials, but the dissociation is now planar and constrained to the (111) slip plane of the dislocation (Figs. 3 ▶ *c* and 3 ▶ *d*).

The analysis of the CXD pattern is less straightforward in this case than for a screw dislocation because of the strain component normal to the slip plane. We use Cartesian coordinates *x*, *y*, *z* so that the *z* axis is along the dislocation line **t** and the *x* axis is along the Burgers vector **b** (the *y* axis is along a third direction **b** × **t**). In the approximation of an isotropic and infinite material, the symmetry of the problem constrains the displacement field in the *xy* plane and it is independent of *z*. Furthermore, an analytical expression can be derived (Hull & Bacon, 2001 ▶; Hirth & Lothe, 1968 ▶): 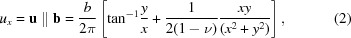


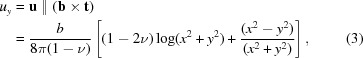
where ν is the Poisson ratio. This analytical displacement field is injected into the perfect nanocrystal as the initial state of the edge dislocation before relaxation. The *u_x_* component of the atomic displacement field is shown in Figs. 3 ▶(*a*) and 3 ▶(*c*) for the initial and relaxed configurations, respectively. Similarly to the case of the screw dislocation, it is equal to ±*b*/2 for a perfect edge dislocation. Upon relaxation it slightly increases owing to the dissociation into partials and to the contraction of surface atoms. For atoms within the (111) stacking fault ribbon *u_x_* = *b*/4. From equations (2) and (3), one can easily understand that complete invisibility of an edge dislocation may only be achieved when **g**·**b** = 0 and **g**·(**b** × **t**) = 0, satisfied only if **g** is parallel to the dislocation line.

As illustrated in Fig. 3 ▶(*e*), when the diffraction vector fulfils this invisibility condition, the dislocation indeed remains invisible and the resulting CXD pattern is similar to that of a perfect crystal. As revealed by the intensity profile along the [111] direction, dissociation of the dislocation (Fig. 3 ▶ *g*) results in the appearance of intense fringes along [111] with twice the period of the crystal finite-size fringes. As shown in the previous section, this is clear evidence of the presence of a stacking fault in the (111) plane located at the centre of the crystallite. In the vicinity of **g** = 22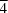, the invisibility condition is not strictly fulfilled, resulting in a large decrease of the maximum intensity of the central spot (around 35%, Fig. 3 ▶ *f*). However, in such diffraction conditions, only displacements parallel to the dislocation line can be detected. They are not strictly equal to zero when the dislocation is relaxed, but they remain very limited and the effect produced by the dissociation on the calculated CXD pattern remains relatively weak. The conditions **g**·**b** = 0 with **g** not parallel to **t** are more suited to show the effect of the dissociation. In these conditions (Fig. 3 ▶ *h*), a perfect dislocation yields a CXD pattern elongated along **b** with a strong decrease of intensity of the Bragg spot (40% of the perfect crystal), consistent with the fact that this diffraction condition is sensitive to the displacements in the planes perpendicular to the dislocation line (Hull & Bacon, 2001 ▶; Wiliams & Carter, 1996 ▶). The CXD pattern obtained for the dissociated dislocation (Fig. 3 ▶ *i* and 3 ▶ *j*) is very similar to that of a dissociated screw dislocation, with a split of the Bragg peak along **b** and fringes along the [111] direction associated with the (111) stacking fault (Fig. 3 ▶ *j*). Similarly to the screw dislocation, the split of the Bragg peak is not visible for low *h*, *k*, *l* values (*h* + *k* + l < 4), which only induce an elongation along **b**. The correlation between the intensity and spacing of Bragg spots and the crystal SFE is addressed in more detail in §3.3.

When **g**||**b** (Figs. 3 ▶ *k* and 3 ▶ *l*), as in the case of a screw dislocation, an edge dislocation produces a strong and characteristic signature, but the effect of dissociation is not as significant. Close to the Bragg position one can notice the elongation of the Bragg spot intensity along **b** for perfect and dissociated dislocations. The effect of dissociation is reflected by an increase of the Bragg spot intensity by a factor of two during relaxation. Both perfect and dissociated dislocations also induce intense fringes along the [111] direction, with an apparent doubling of the fringes’ period. This doubling of the period has also been reported by Wilson (1952 ▶, 1955 ▶) and Gailhanou & Roussel (2013 ▶) in the case of a perfect screw dislocation. It is not related to a (111) stacking fault since it is observed for both perfect and dissociated dislocations. For **g**||**b**_p_ (Figs. 3 ▶ *n* and 3 ▶ *o*), similar fringes along [111] and an elongation along **b** can be observed for both perfect and dissociated dislocations. A more surprising result is the vanishing intensity at the exact Bragg peak position, probably related to the π/2 phase jump induced by the dislocation for the 2
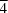
2 reflection. As in the **g**||**b** case, the intensity of the central spots increases by a factor of three during relaxation.

For any other selected diffraction vector, the calculated CXD pattern results in two clear and identifiable effects: a splitting or at least an elongation along **b** and intense fringes along [111] (*i.e.* the direction perpendicular to the dissociation plane).

### 3.3. Stacking faults   

Similarly to dislocations, stacking faults induce a global shift of one part of the crystal with respect to another and thus appear as phase defects in diffraction. But, while dislocations induce a long-distance heterogeneous strain field, elastic strain caused by a stacking fault remains limited to the vicinity of the fault (Hirth & Lothe, 1968 ▶). According to equation (1), CXD is sensitive to the displacement field, even in the absence of elastic strain, and in fact the stacking fault is the case that can produce the maximum interference contrast. The relatively simple signature on CXD patterns combined with their frequent occurrence in nanowires with low SFE (one-dimensional systems) has already motivated numerous studies of such materials using CXD (Chamard *et al.*, 2008 ▶; Favre-Nicolin *et al.*, 2010 ▶; Jacques *et al.*, 2013 ▶): it has been used to evaluate the number of stacking faults in an InSb pillar (Jacques *et al.*, 2013 ▶) and to get useful information about the fault sequence in a GaAs/GaP nanowire (Favre-Nicolin *et al.*, 2010 ▶). While CXD has been mostly used to study systems with no or very few crystal defects, these studies demonstrate that it can be used efficiently on systems with multiple defects. This opens the perspective to apply the technique to a wider range of systems, even if the case of multiple defects is so far limited to one-dimensional systems. In the present paper we deal with the case of stacking faults in three-dimensional systems. Stacking faults are fairly common in f.c.c. metals and usually occur in {111} crystallographic planes.

Let us start with the simple case of a stacking fault completely separating the crystal into two parts either side of a (111) plane. The phase jump Δϕ across the stacking fault can be expressed as 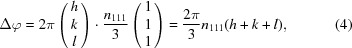
where *n*_111_ is the number of faulted planes. If it is a multiple of 3, Δϕ is a multiple of 2π for any Bragg reflection and it is impossible to show the fault in diffraction, unless the volume of the faulted part becomes comparable to that of the rest of the crystal.

**Figure 4:**
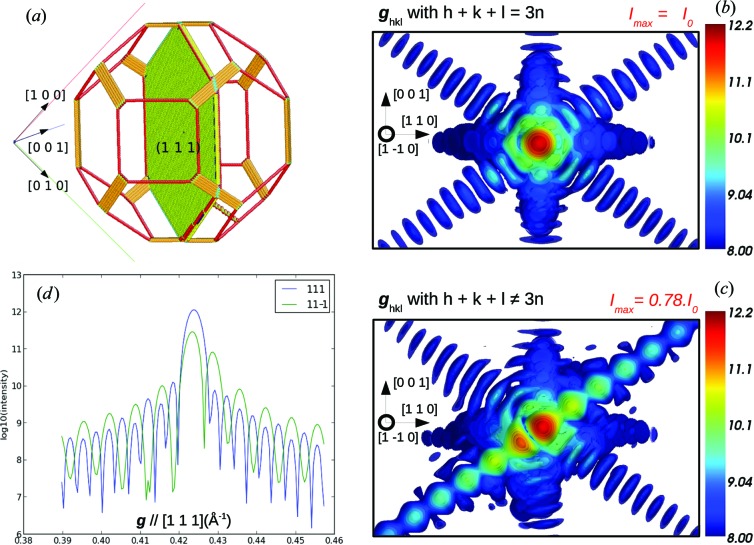
(*a*) (111) Stacking fault in a silver crystal with a Wulff geometry induced by the complete relaxation of a perfect edge line dislocation. (*b*) Corresponding CXD pattern when **g** fulfils the extinction conditions, *i.e. h* + *k* + *l* = 3*n* (**g** = 111). (*c*) The same CXD pattern when *h* + *k* + *l* ≠ 3*n* (**g** = 11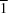). (*d*) Intensity along [111] for both cases (log scale). The selected area of the reciprocal space is kept to the same value in all figures and is equal to 0.045 × 0.0675 Å^−1^.

A stacking fault is created by the insertion or the removal of a close-packed {111} layer in the crystal. The removal of a plane is called an intrinsic stacking fault, whereas the insertion of a layer is called an extrinsic stacking fault. If the stacking fault results from the dissociation of a perfect dislocation, it is necessarily intrinsic (*n*_111_ = 2). Close to a Bragg position, the *h*, *k*, *l* values can be approximated by the integer values of the Bragg position. Depending on the selected diffraction vector, only two cases can occur. When *h* + *k* + *l* = 3*n*, the resulting phase jump is a multiple of 2π and the stacking fault remains invisible (Fig. 4 ▶ *b*). This invisibility condition can be exploited to hide a particular type of stacking fault and instead highlight elastic strain and other defects (Favre-Nicolin *et al.*, 2010 ▶). When *h* + *k* + *l* ≠ 3*n*, the stacking fault causes a phase shift of ±2π/3 between the two parts of the crystal, inducing a strong signature in the diffraction pattern. The intensity in the vicinity of the Bragg position **g** can then be expressed as follows: 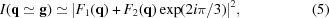
where *F*_1_ and *F*_2_ are the structure factors of the crystal parts on either side of the stacking fault. At the exact Bragg position, *F*_1_ and *F*_2_ are essentially proportional to the respective volume fractions *x* and 1 − *x* of unfaulted material either side of the stacking fault and 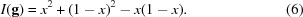
Destructive interference is maximal when the two volumes are equal: the intensity is then a quarter of the intensity diffracted by the perfect crystal, which means that the best contrast is obtained when the stacking fault is located in the middle of the volume.

The complete picture of the vicinity of the Bragg position (**q** ≃ **g**) is obtained with *PyNX* calculations performed on our model crystal after introduction of a traversing stacking fault passing through the centre (Fig. 4 ▶ *a*). It confirms that the stacking fault is invisible on the 111 reflection (*h* + *k* + *l* = 3*n*) (Fig. 4 ▶ *b*), while it has a clear signature on the 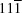 reflection (*h* + *k* + *l* ≠ 3*n*) (Fig. 4 ▶ *c*). The intensity at the exact 11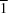 Bragg position roughly equals one-quarter of the intensity at the exact 111 Bragg position, as predicted above. The most characteristic signature of the stacking fault is the reinforcement of the intensity on the streak along [111], with a modification of its fringes, while fringes along other directions are barely changed. Here the modification of the fringes is essentially a doubling of the period, which is a consequence of the stacking fault being in the middle of the crystal.

Regarding experimental matters, it is also important to be able to distinguish between intrinsic and extrinsic stacking faults. It is a well known result that the elastic diffuse scattering from dislocation loops is sensitive to the vacancy (presence of an intrinsic stacking fault) or interstitial (presence of an extrinsic stacking fault) character of a partial dislocation loop (Ehrhart *et al.*, 1982 ▶). Here we perform the calculation around the 220 reflection (*h* + *k* + *l* ≠ 3*n*). For an intrinsic stacking fault, as illustrated in Fig. 4 ▶(*b*), the satellite spot (weakest part of the split Bragg peak) is located in the lower **q** values with respect to the Bragg peak position. For an extrinsic stacking fault (not shown here), this same satellite spot is located in the positive **q** values with respect to the Bragg position. These results are in good agreement with previous calculations on dislocation loops and stacking faults (Ehrhart *et al.*, 1982 ▶; Nordlund *et al.*, 2000 ▶).

**Figure 5:**
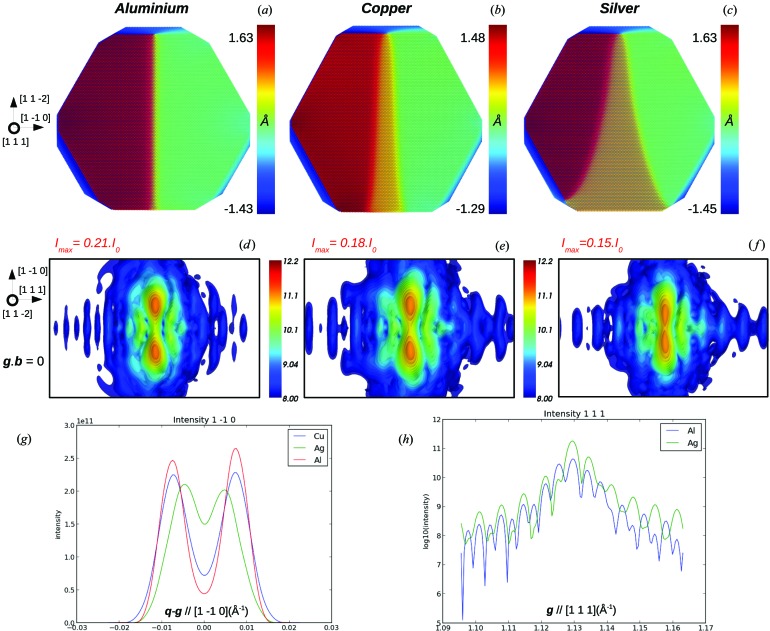
Dissociated edge dislocations in a 30 × 30 × 30 nm crystal and corresponding displacement field (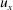 component) for aluminium (*a*), copper (*b*) and silver (*c*) crystals with a Wulff geometry. (*d*)–(*f*) Corresponding CXD patterns with **g**·**b** = 0 (**g** = 224). (*g*) and (*h*) Intensity profiles along [1
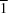
0] and [111] (logarithmic scale). The selected area of the reciprocal space is kept to the same value in all figures and is equal to 0.045 × 0.0675 Å^−1^.

Traversing stacking faults are not the only common case in nanocrystals: as seen above, dissociated dislocations can stabilize in ribbon-shaped stacking faults, because of the competition between SFE and repulsive forces between the partials. It is interesting to see if one can get an idea of the extension of a single stacking fault from a CXD measurement. For a given material, the ability of a perfect dislocation to dissociate and produce a stacking fault is influenced by two main parameters: its stacking fault energy γ_s_ and its shear modulus μ. The dissociation length of a dislocation is controlled by the adimensional material parameter γ_s_/μ*b*_p_, where *b*_p_ is the modulus of the partial Burgers vector of the dislocation (Chassagne *et al.*, 2011 ▶). Materials with a low γ_s_/μ*b*_p_ value have widely dissociated dislocations with a high constriction stress, while the occurrence of dissociated dislocations or stacking faults is less frequent in materials with a high γ_s_/μ*b*_p_. Calculations were performed on five different f.c.c. metals with similar size and shape (Fig. 5 ▶, gold and nickel are not shown) and SFEs ranging from 17.8 mJ m^−2^ (silver) to 149.3 mJ m^−2^ (aluminium) (Cockayne *et al.*, 1971 ▶). Values given by EAM potentials and experiments are reported in Table 1 ▶. They are in very good agreement, except for the case of gold, for which the discrepancy between EAM and experimental values is close to 25%. We use the SFE given by the EAM potentials to calculate the parameter γ_s_/μ*b*_p_, whose values are reported in Table 1 ▶. As illustrated in Figs. 5 ▶(*a*)–5 ▶(*c*), the dissociation length obtained upon relaxation (1600 relaxation steps) decreases consistently when γ_s_/μ*b*_p_ increases: the dislocation is widely dissociated in silver, which has the lowest γ_s_/μ*b*_p_, whereas the dissociation remains very limited in aluminium (highest γ_s_/μ*b*_p_).

When looking at the CXD patterns (Figs. 5 ▶ *d*–5 ▶ *f*) and the intensity profile along [1
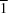
0] (Fig. 5 ▶ *g*), one observes the inverse phenomena: a narrow stacking fault induces a large splitting distance (*i.e.* the distance between the maxima of intensity of the split Bragg peak) with intense maxima of intensity, a low minimum of intensity at the Bragg position and a large splitting distance of the Bragg peaks (Figs. 5 ▶ *d*–5 ▶ *g*), whereas a wide stacking fault induces a weak splitting, with low maxima of intensity, low intensity drop in Bragg position and a small splitting distance of the Bragg peaks. One can also notice the increasing intensity of the [111] fringes and the decreasing distance between the maxima of intensity along [1
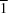
0] as the stacking fault spreads into the crystallite. Copper, gold and nickel have similar γ_s_/μ*b*_p_ values and the resulting dislocation dissociation lengths upon relaxation for these three materials are hence rather close. The case of nickel is quite interesting since it has an SFE similar to that of aluminium; however, its high shear modulus allows it to attain a dissociation length equivalent to that obtained for copper. This illustrates the influence of both parameters on the occurrence of stacking faults. Calculations of CXD patterns for nickel and gold (not shown here) logically lead to results very similar to the case of copper. Regarding experimental matters, it is then a safe assumption to expect the same kind of structural defects in these three f.c.c. metals, and as a result the calculations presented for copper in this study can also be used as a reference for experimental work on gold or nickel crystallites.

**Figure 6:**
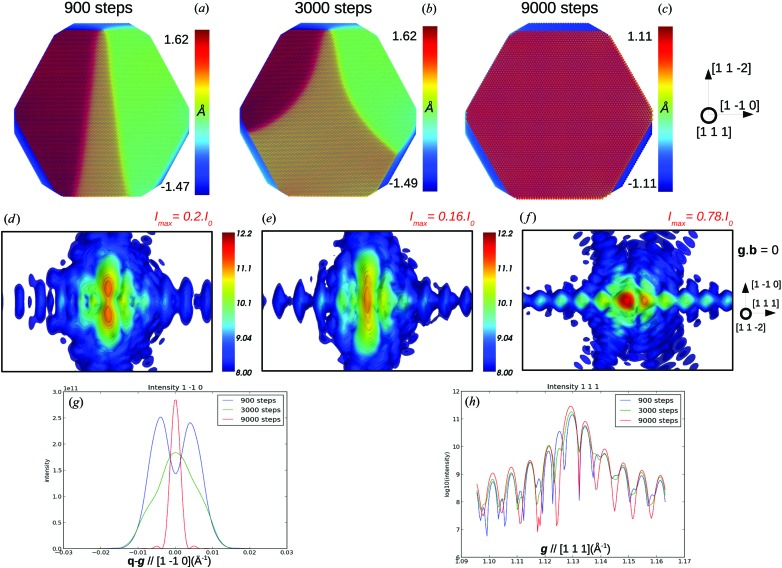
Relaxation for a crystal with a low SFE (silver). *u_x_* component of the atomic displacement after 900 relaxation steps (*a*), 3000 relaxation steps (*b*) and full relaxation (*c*). (*d*)–(*f*) Corresponding CXD pattern for **g**·**b** = 0 (**g** = 224). (*g*)–(*h*) Intensity along [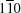] and [111] (log scale). The selected area of the reciprocal space is kept to the same value in all figures and is equal to 0.045 × 0.0675 Å^−1^.

From these first conclusions, some complementary calculations on the relaxation of systems with low SFE such as silver were performed. During the first steps of relaxation (Figs. 6 ▶ *a* and 6 ▶ *d*), the stacking fault remains rather narrow. For **g**·** b** = 0, both partials and the stacking fault display a strong signature on the CXD pattern, with, respectively, a splitting of the Bragg peak along **b** and intense fringes along [111]. After 3000 relaxation steps, the stacking fault continues to spread and the splitting of the Bragg reflection cannot be observed any longer, while the [111] fringes become more intense. As the stacking fault extends, the intensity at the Bragg position increases, while the global maximum of intensity steadily decreases, and so does the splitting of the Bragg reflection (Figs. 6 ▶ *b*, 6 ▶ *e* and 6 ▶ *g*). At this stage of relaxation, the inhomogeneous strain around the dislocation induces a very low maximum of intensity on the CXD pattern (Figs. 6 ▶ *e* and 6 ▶ *g*). During the final steps of relaxation, the stacking fault continues to spread until it emerges on one of the crystal facets and the signature of the Shockley partials (*i.e.* splitting of the Bragg peak along **b**) completely vanishes, while the intensity of the [111] fringes increases with the width of the stacking fault (Fig. 6 ▶ *h*). One can also notice the sharp increase of the maximum intensity which coincides with the disappearance of the Shockley partials from the nanocrystal. One can assume that the rather large inhomogeneous strain around the partials during the dissociation (Figs. 6 ▶ *a* and 6 ▶ *b*) results in a drop of intensity during the relaxation. As the partials leave the crystal, the strain around the stacking fault is weak and with a very limited extent (restrained to the two faulted planes of the intrinsic stacking fault) (Fig. 6 ▶ *c*), resulting in a larger intensity close to the Bragg position.

### 3.4. Frank loop   

**Figure 7:**
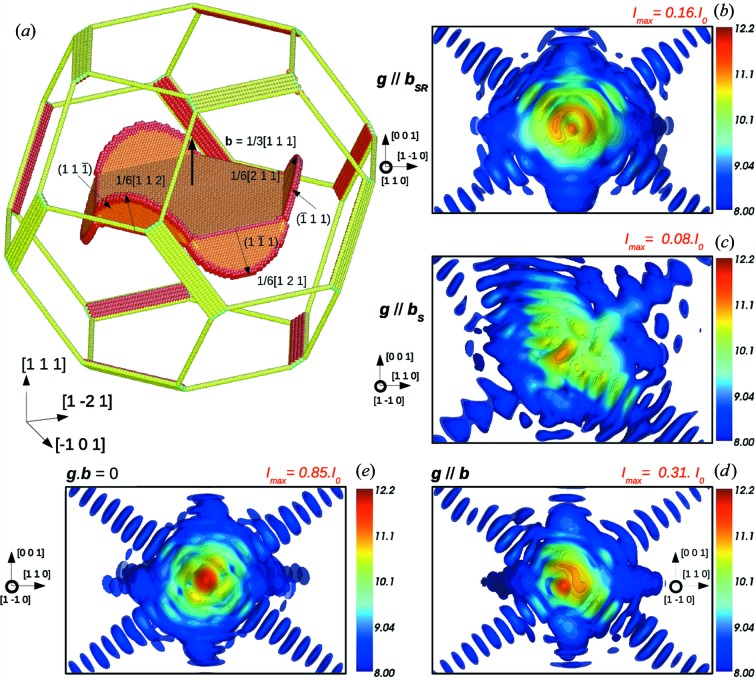
(*a*) Relaxed Frank dislocation loop with **b** = 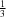
[111] in the centre of a 30 × 30 × 30 nm Wulff silver crystal. The colour code represents the coordination number, such that only the defective atoms and nanocrystal edges are shown. Calculated CXD patterns when **g**||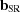 (**g** = 220) (*b*), when **g**||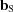 (**g** = 224) (*c*), when **g**||**b** (**g** = 111) (*d*) and when **g**·**b** = 0 (**g** = 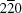) (*e*). The selected area of the reciprocal space is kept to the same value in all figures and is equal to 0.045 × 0.0675 Å^−1^.

A Frank partial dislocation is formed as the boundary of a fault formed by inserting or removing a close-packed {111} layer of atoms in a perfect crystal. Geometrically, the Frank intrinsic stacking fault is identical to the intrinsic fault produced by the dissociation of a perfect dislocation, except that the bounding partial is different. An intrinsic Frank loop is often called a vacancy Frank loop, whereas an extrinsic Frank loop can be referred to as an interstitial Frank loop. The Burgers vector of a Frank loop is perpendicular to the {111} fault plane, with a magnitude equal to the interplanar spacing, *i.e.* **b** is of type 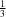
〈111〉. Here, an extrinsic Frank loop with **b** = 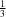
[111] is introduced in a 30 × 30 × 30 nm silver nanocrystal with a Wulff shape (Fig. 7 ▶ *a*).

During relaxation, the Frank partial dissociates into a low-energy, so-called stair-rod, dislocation (Hull & Bacon, 2001 ▶) and a Shockley partial on an intersecting {111} plane according to a reaction of the type 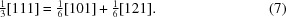
The hexagonal Frank loop with Burgers vector 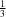
[111] can dissociate to produce a stair rod along each edge and a Shockley partial on the three inclined {111} planes as illustrated in Fig. 7 ▶(*a*).

Calculations of diffuse scattering performed on perfect (prismatic) and partial (Frank) dislocation loops in f.c.c. metals (Ehrhart *et al.*, 1982 ▶) and semiconductors (Nordlund *et al.*, 2000 ▶) have already provided a very accurate picture of the scattering that can be expected from such defects. The Huang diffuse scattering of perfect and Frank dislocation loops has also been studied experimentally by Larson & Schmatz (1980 ▶) and Larson & Young (1987 ▶). They have demonstrated that Huang diffuse scattering can be used to determine the vacancy or interstitial character of a loop, to estimate the relative proportion of this type of loop in a given population, and to estimate their size. It is shown by Nordlund *et al.* (2000 ▶) that the general features observed in diffuse scattering patterns are mostly independent of the choice of the Bragg peak. We demonstrate in the following that in the case of CXD, the choice of the Bragg reflection is essential to show the characteristic signature of a Frank or a prismatic dislocation loop.

A Frank loop is a pure edge dislocation since the Burgers vector is always perpendicular to the dislocation line. Contrary to the case of a straight edge dislocation there is no diffraction condition where **g**·**u** = 0 for all the loop edges [*i.e.* **g**·**b** = 0 AND **g**·(**b** × **t**) = 0]. This particular case can be used to distinguish a Frank loop from a straight dislocation when analysing CXD patterns.

As in the case of a straight dislocation line (§[Sec sec3.2]3.2), **g**·**b** = 0 is a partial extinction condition, since it ignores the part of the displacement parallel to the Burgers vector, such that little perturbation is observed around these reflections (Fig. 7 ▶ *e*). The other part of the displacement field and the relaxation in stair rods and Shockley partials are responsible for the weak reduction of intensity of the central peak (85% of the perfect crystal) and the weak distortions of the pattern visible in Fig. 7 ▶(*e*).

The case **g**||**b** at the end of the relaxation (Fig. 7 ▶ *d*) also produces some interesting results, with a drastic reduction of intensity of the central spot (30% of the perfect crystal) and the appearance of a satellite spot along the [111] direction. This reduction in intensity is obviously related to the presence of the loop in the centre of the volume. In agreement with the invisibility conditions for a stacking fault detailed in §[Sec sec3.3]3.3, the characteristic signature of a (111) stacking fault, *i.e.* fringes along [111], is not visible on the CXD pattern in this case. This particular reflection is also well suited to determine the interstitial or vacancy character of the Frank loop. As shown in Fig. 7 ▶(*d*), the scattering is more intense for the high **q** values (presence of a satellite peak) with respect to the theoretical Bragg position. This distribution of the scattering is expected for an interstitial Frank loop and is in good agreement with the results of Ehrhart *et al.* (1982 ▶) and Nordlund *et al.* (2000 ▶). In the case of a vacancy Frank loop and for this particular reflection (not shown here), the satellite peak is located in the lower **q** values with respect to the theoretical Bragg position.

As illustrated in Fig. 7 ▶(*b*), the dissociation in Shockley and stair-rod partials induces a very characteristic signature on CXD patterns when the diffraction vector is parallel to a 〈110〉 direction (Fig. 7 ▶ *b*) (but not perpendicular to **b**, *i.e.* only the *hh*0, *h*0*h* and 0*hh* reflections with *h* even can be used), corresponding to the Burgers vector of a partial stair rod. The pattern then looks fairly similar to that of a screw dislocation, with a ring-shaped pattern oriented along **b**_SR_ = 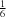
〈110〉 (where ***b***_SR_ is the Burgers vector of the stair-rod dislocation). This kind of pattern is not observed when the loop is not dissociated and is a clear indication of the formation of a stair-rod dislocation during relaxation.

When looking at the other set of partials, *i.e.* when **g** is parallel to one of the Shockley partials [**g**||**b**_S_, **g** = 224 (Fig. 7 ▶ *c*), where **b**_S_ is the Burgers vector of the Shockley partial dislocation], the resulting CXD pattern is very disturbed at the end of relaxation, with intense fringes along [111] and an elongated central spot with very low intensity in comparison to a perfect crystal (only 8% of the Bragg peak intensity). During the first stages of relaxation, the intensity of the central spot is similar to that of a perfect crystal, and only the fringes along [111] indicate the presence of a defect in the crystal. Hence these particular Bragg conditions appear particularly well suited to show the dissociation of the Frank partial in its intersecting slip planes.

### 3.5. Prismatic loop   

**Figure 8:**
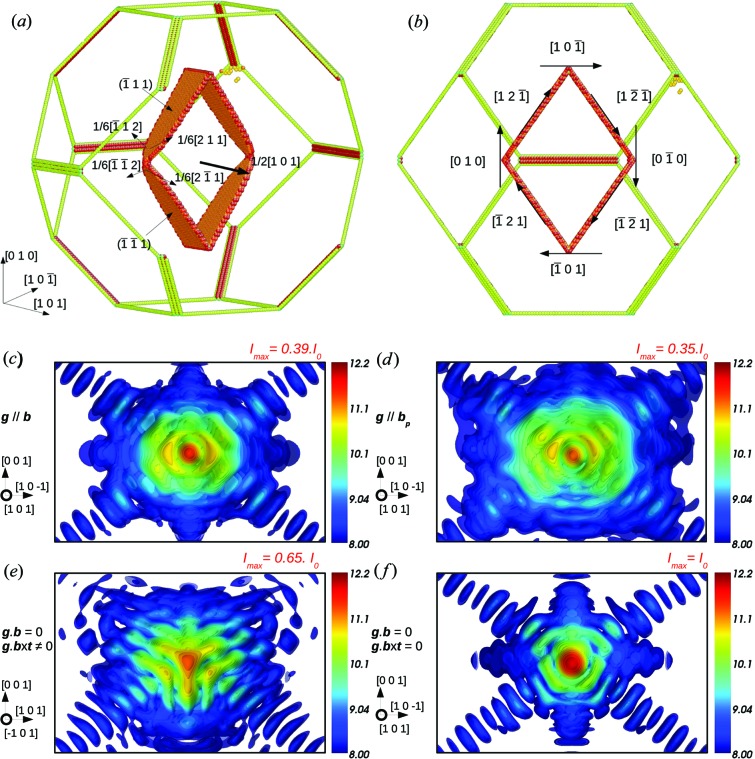
(*a*) Relaxed prismatic dislocation loop with **b** = 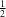
[101] at the centre of a 30 × 30 × 30 nm Wulff copper crystal. The colour code represents the coordination number, such that only the defective atoms and nanocrystal edges are shown. The loop decomposes into partial dislocations in its (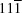) and (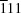) slip planes. (*b*) The same dislocation loop viewed along the [101] direction. Calculated CXD pattern for **g**||**b** (**g** = 202) (*c*), **g**||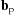 (**g** = 422) (*d*), **g**·**b** = 0 and **g**·(**b** × **t**) ≠ 0 (**g** = 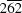) (*e*), and **g**·**b** = 0 and **g**·(**b** × **t**) = 0 (**g** = 020) (*f*). The selected area of the reciprocal space is kept to the same value in all figures and is equal to 0.045 × 0.0675 Å^−1^.

A prismatic dislocation loop has a Burgers vector not contained in the plane of the loop. We introduce a prismatic loop at the centre of a 30 × 30 × 30 nm copper crystal with a Wulff geometry. The Burgers vector **b** = 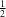
[101] decomposes during relaxation into partial dislocations in its (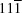) and (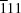) slip planes, as illustrated in Fig. 8 ▶(*a*). Since the Burgers vector is perpendicular to the dislocation line, the loop edges are pure edge dislocations and the invisibility criteria, *i.e.* **g**·**b** = 0 and **g**·(**b** × **t**) = 0, described in §3.2 apply for this type of defect. However, as in the Frank loop case, since the loop edges are not all aligned, there are always segments of the dislocation loop where **g**·(**b** × **t**) ≠ 0, which produces a visible effect on the CXD pattern. But, when **g**·**b** = 0 and **g**·(**b** × **t**) = 0 for two opposite segments of the loop (for instance **g** = 020) (Fig. 8 ▶ *f*), the signature of the prismatic loop on CXD patterns is very faint, and the intensity of the Bragg spot is similar to the case of the perfect crystal, with no elongation in any particular direction. When **g**·**b** = 0 but **g**·(**b** × **t**) ≠ 0, *i.e.* **g** is not parallel to any segment of the loop [the case **g** = 2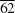 is shown in Fig. 8 ▶(*e*)], the prismatic loop induces some perturbation in the CXD pattern, which is expected since such conditions do not lead to a complete extinction for an edge dislocation. The central spot intensity slightly decreases (65% of that of a perfect crystal) and the diffraction pattern is elongated in the (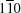) plane along the [111], [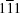] and [001] directions. We now focus on diffraction conditions where the prismatic loop should produce a strong and characteristic signature, *i.e.* **g**||**b**. As shown in Fig. 8 ▶(*c*) (**g** = 202), one can observe a hexagonal-shaped pattern with an elongation along the Burgers vector direction **b** = 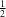
[101] and a strong decrease of the intensity of the central spot (by half compared to the perfect crystal). One can also notice the increased intensity of the [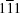] and [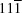] fringes, due to the stacking faults in the dissociated loop edges. Similarly to an edge dislocation, the conditions when **g**||**b**_p_, for instance **g** = 422 (Fig. 8 ▶ *d*), also produce a characteristic signature (Fig. 8 ▶ *e*). The resulting diffraction pattern is similar to the case **g**||**b** (Fig. 8 ▶ *c*) with a hexagonal-shaped pattern elongated along **b** and a reduction of the central spot intensity by a factor of three. Finally, for a general diffraction vector **g** the defect signature can clearly be identified on the CXD pattern, but its intensity is generally lower than for the particular cases **g**||**b** and **g**||**b**_p_. Additionally, the hexagonal-shaped pattern is slightly disoriented with respect to **b**.

In conclusion to this section, similarly to simple dislocation lines and stacking faults, Frank and prismatic loops produce a characteristic signature strongly influenced by the choice of the diffraction vector and the invisibility conditions. The main difference between a dislocation loop and a line dislocation lies in the choice of the diffraction conditions to show such defects. While for the latter the case **g**·**b** = 0 is an appropriate choice to show dissociation, this condition is less adapted to dislocation loops since it will hide their characteristic signature. However, we will see in the last section that the proper use of these invisibility conditions turns out to be particularly useful to determine the Burgers vector of any kind of dislocation.

This study of simple and ideal cases of single defects drives us to a simple conclusion: a given crystalline defect has a characteristic signature, which can be identified and interpreted using coherent X-ray diffraction. Equally important is the influence of the diffraction vector on the resulting CXD pattern, and the need to select the appropriate vector in order to highlight or hide the signature of a given crystal defect. One has to keep in mind that particular cases detailed throughout this study are not always the best suited for all types of crystalline defects. These considerations should be useful in order to select the best experimental conditions to show a given crystalline defect. Additionally, as illustrated in the next section, these simple cases can be used to understand and interpret CXD patterns from more complex and realistic structures. An overview of the cases detailed throughout this study is presented in Table 2 ▶, which highlights the best diffraction conditions to show each type of crystalline defect.

### 3.6. Influence of the crystal size and shape   

**Figure 9:**
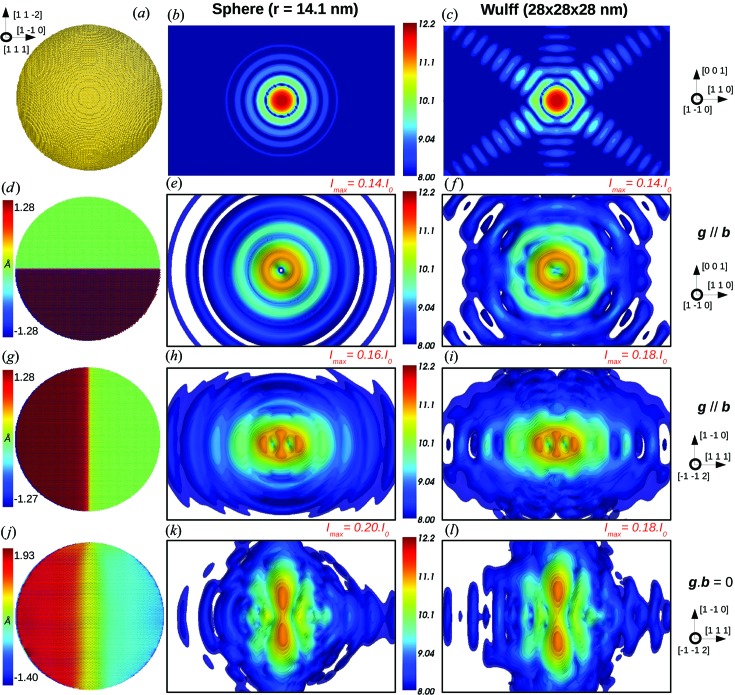
(*a*) Defect-free copper spherical crystal with *r* = 14.1 nm. Corresponding CXD patterns with **g**||**b** (**g** = 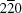) for the sphere (*b*) and the reference copper crystal in a Wulff geometry (*c*). (*d*) Perfect screw dislocation with **b** = 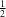
[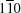] at the centre of the section in the same spherical crystal. The colour scale shows the 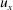 component of the atomic displacement field. Corresponding CXD patterns with **g**||**b** (**g** = 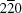) for a sphere (*e*) and a Wulff crystal (*f*). (*g*) Perfect edge dislocation with **b** = 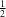
[1
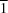
0] in the same crystal. Corresponding CXD patterns with **g**||**b** (**g** = 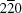) for a sphere (*h*) and a Wulff crystal (*i*). (*j*) Dissociation of the perfect dislocation into two Shockley partials in the (111) plane with 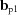 = 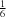
[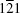] and 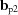 = 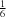
[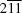] in the same crystal. Corresponding CXD patterns with **g**·**b** = 0 (**g** = 224) for a sphere (*k*) and a Wulff crystal (*l*). The selected area of the reciprocal space is kept to the same value in all figures and is equal to 0.045 × 0.0675 Å^−1^.

The cases detailed in §§3.1–3.5 share the same geometry with a single defect introduced at the centre of a Wulff crystal. However, the position of the dislocation and the boundary conditions of the crystal might have a considerable influence on the defect signature, and their effect is now investigated. To study the effect of the crystal shape, we compare the results obtained with a crystal of Wulff geometry with a spherical crystal. We simulated a sphere of copper with radius *r* = 14.1 nm (corresponding to 1.2 × 10^6^ atoms, a number similar to the reference crystal), at the centre of which we introduce a dislocation line of pure screw or pure edge character, with Burgers vector **b** = 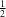
[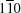]. Similarly to what has been observed with the Wulff geometry, the perfect screw dislocation dissociates during relaxation into two sets of two Shockley partials in its two {111} slip planes, while the edge dislocation dissociates in the (111) plane only (Fig. 9 ▶ *j*). As illustrated in Figs. 9 ▶(*d*) and 9 ▶(*g*), the *u_x_* component of the displacement field is very similar to the one obtained for a Wulff geometry. In both cases it is exactly equal to ±*b*/2. The only differences that can be expected in the calculated CXD patterns should be related to the nanocrystal shape. In the case of perfect crystals, the influence of the shape is seen in the form factor: instead of streaked fringes along the facet directions, one observes spherical fringes, and the shape of the central spot also reveals the geometry (Figs. 9 ▶ *b* and 9 ▶ *c*). Such details are easily shown experimentally with decent statistics. To examine the case of faulted crystals, we choose a diffraction vector parallel to the Burgers vector (**g** = 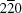). As illustrated in Fig. 9 ▶(*e*), the perfect screw dislocation still yields a ring-shaped pattern with its axis along the Burgers vector direction. The crystal shape only affects the distribution of intensity in the ring (Figs. 9 ▶ *e* and 9 ▶ *f*). For the perfect edge dislocation, similar conclusions are drawn, and the calculated CXD patterns displays the same features as have been observed for the Wulff crystal, such as fringes along the [111] direction and the elongation of the Bragg peak along **b**. The distribution of intensity is very similar for both Wulff and spherical crystallites (Figs. 9 ▶ *h* and 9 ▶ *i*).

The edge and screw dislocations are not stable in a spherical crystallite and the Shockley partials tend to leave the crystallite during relaxation. To make relevant comparisons between relaxed dislocations in the sphere and the Wulff crystallites, the relaxation is stopped after the same number of steps in both configurations (typically 1600 steps for the copper nanocrystal) before the disappearance of the partials from the crystallite (Fig. 9 ▶ *j*). Additionally, the contraction of the surface atoms towards the bulk during relaxation is strongly affected by the change of geometry. For these two reasons, the obtained values of the *u_x_* component of the atomic displacement field (Fig. 9 ▶ *j*) differ from the ones obtained in the Wulff geometry (§[Sec sec3.2]3.2).

We use the extinction condition **g**·**b** = 0 (**g** = 224) to show the effect of dissociation. The Bragg peak splits along the Burgers vector direction, and fringes along the normal to the stacking fault (**n** = [111]) are clearly seen, even though the crystal does not have (111) facets (Fig. 9 ▶ *k*). Another interesting observation is the similarity of the ratio *I*_defect_/*I*_0_ between the two crystallites for all types of defects.

From these examples, one can conclude that the boundary conditions have only limited influence on CXD patterns. While the shape determines the form factor of the Bragg reflection, yielding for instance strong fringes in faceted crystals, the shape and intensity distribution of the features induced by the defects, generally close to the Bragg position, are only marginally affected. It is important to notice that a logarithmic scale, and therefore a few decades of dynamical range in the data, are needed to characterize the form factor, whereas the defects have an obvious impact on the central part of the pattern if the Bragg reflection is well chosen.

The effect of the crystallite size has also been investigated by comparing the obtained CXD patterns of Wulff shaped crystals with sizes ranging from 5 to 60 nm. While this would be a simple scaling exercise in a continuous description of matter such as FEM, here the problem is not invariant because of the fundamental size of the defect, given by the modulus of the Burgers vector. Of course, we evidence in the diffraction patterns the scaling of the form factor in proportion to the change of size of the crystal. But, one might expect a significant effect related to the change of ratio between crystal shape and defect size. However, no significant size effects are seen on the signatures of the defects, regardless of the type of defect and the chosen Bragg reflection, in the range of sizes explored. This suggests that we are still in a size range in which a continuous description of matter would be valid, providing a sufficiently good continuous description of the defect and its strain field. An important consequence of the weak influence of size and shape of the crystal containing the defect is that the results presented above can be generally applied to a wide range of size and shapes of f.c.c. crystals. This is particularly useful since samples may contain many crystals of the same materials with a wide range of size and shapes, depending on the processing route (in particular in the case of dewetting; Beutier *et al.*, 2013 ▶; Mordehai, Lee *et al.*, 2011 ▶; Mordehai, Kazakevich *et al.*, 2011 ▶).

### 3.7. Influence of the defect position   

**Figure 10:**
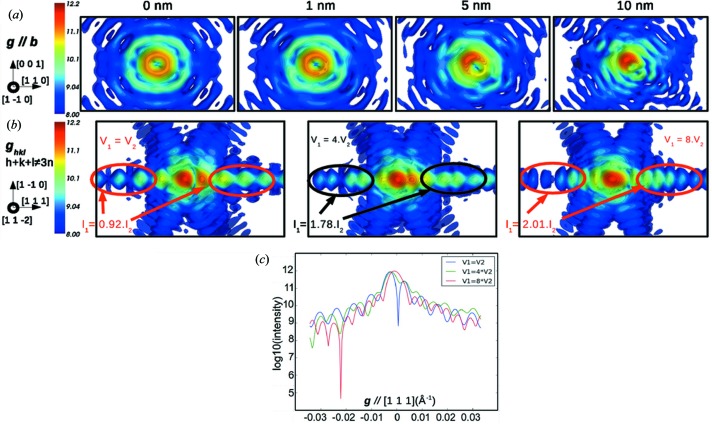
(*a*) Effect of the position of a perfect screw dislocation in a 30 × 30 × 30 nm copper crystal in a Wulff geometry for **g**||**b** (**g** = 2
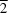
0). In the vicinity of the crystal centre the intensity distribution is altered, and as the dislocation moves towards an edge of the crystal its characteristic signature completely vanishes. (*b*) Effect of the position of a stacking fault in a 30 × 30 × 30 nm copper crystal in a Wulff geometry for **g** = 11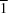. The stacking fault position strongly affects the fringe intensity and period, and the intensity and splitting of the Bragg reflection. (*c*) Intensity along [111] for different positions of the stacking fault in the crystallite. The selected area of the reciprocal space is kept to the same value in all figures and is equal to 0.045 × 0.0675 Å^−1^.

To show the effect of the defect position, we chose to focus on two simple defects: a perfect screw dislocation and a stacking fault, both in a 30 × 30 × 30 nm copper crystal of Wulff shape. The screw dislocation is introduced at several positions in the crystal – 0, 1, 5 and 10 nm away from the centre of the crystal – and the 2
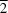
0 reflection is used to probe the dislocation. As illustrated in Fig. 10 ▶(*a*), the displacement of the dislocation line induces a considerable effect on the intensity distribution of the calculated diffraction pattern. As the dislocation moves towards the emerging facets of the crystal, the distribution of intensity in reciprocal space becomes highly anisotropic until the ring-shaped pattern vanishes when the dislocation reaches one edge of the crystal. The same results could be obtained for an edge dislocation line in both its perfect and its relaxed states (not shown here).

Another very important consideration is the unstable character of dislocations that are not introduced close to the centre of the crystallite. According to our calculations in the reference crystallite, for a perfect dislocation introduced more than 3 nm away from the centre, the Shockley partials always leave the crystallite during relaxation. One can then assume that the probability of probing dissociated dislocations far away from the crystallite centre in experimental crystals is very low. This strengthens the relevance of our study since most of the calculations are performed with dislocations introduced at the centre of the crystallite.

As seen in §[Sec sec3.3]3.3, a stacking fault introduced at the centre of the reference crystal leads to a splitting of the central spot and intense fringes along the normal to the (111) stacking fault plane, with a doubling of spacing between fringes. When the stacking fault is placed at the centre of the crystal, the two parts of the object that interfere are equal, yielding a symmetric distribution of intensity in the fringes along the [111] direction (see Figs. 10 ▶ *b* and 10 ▶ *c*). A stacking fault off the crystal centre splits the volume into two unequal parts and yields an asymmetric distribution of intensity along the [111] axis. Fig. 10 ▶(*b*) indeed shows that a stacking fault splitting the crystal into two volumes such that *V*_1_ = 4*V*_2_ yields an asymmetry of the [111] fringe intensity distribution, which is further increased when the stacking fault is moved towards an edge of the crystal (*V*_1_ = 8*V*_2_). The intensity at the exact Bragg position can be evaluated and, according to equation (6), as the stacking fault moves away from the centre, the interference becomes less destructive and the Bragg position becomes a peak of intensity again, like for the perfect crystal.

This section confirms that the defect position has a very strong effect on the calculated CXD patterns. This effect increases with the distance between the defect and the centre of the illuminated crystal. In the vicinity of the centre, the intensity distribution is strongly altered, but a given defect can clearly be identified from its signature on the diffraction pattern. However, close to an edge of the crystal, the characteristic signature of a given defect vanishes, and our ability to identify the defect from its signature in reciprocal space becomes questionable.

## 4. Application to a complex case: indentation of a gold nanocrystal   

The study of model systems is very useful to understand and interpret the signature induced by a single defect and to demonstrate the influence of the selection of the diffraction vector on CXD patterns. However, interpretations of the pattern can also be deduced for more complex and realistic configurations of defects, such as the one obtained during plastic indentation of a crystallite. More details con­cerning the dislocation mechanisms during nanoindentation are given by Mordehai, Kazakevich *et al.* (2011 ▶). In the present section, only a few key stages of the indentation process and the corresponding CXD patterns in reciprocal space are detailed. Molecular dynamics simulations with the large-scale atomic/molecular massively parallel simulator (LAMMPS; Plimpton, 1995 ▶) and an Au EAM potential (Grochola *et al.*, 2005 ▶) are used to simulate the indentation of a 12.1 nm gold nanoparticle on a sapphire substrate (Mordehai, Lee *et al.*, 2011 ▶; Mordehai, Kazakevich *et al.*, 2011 ▶).

**Figure 11:**
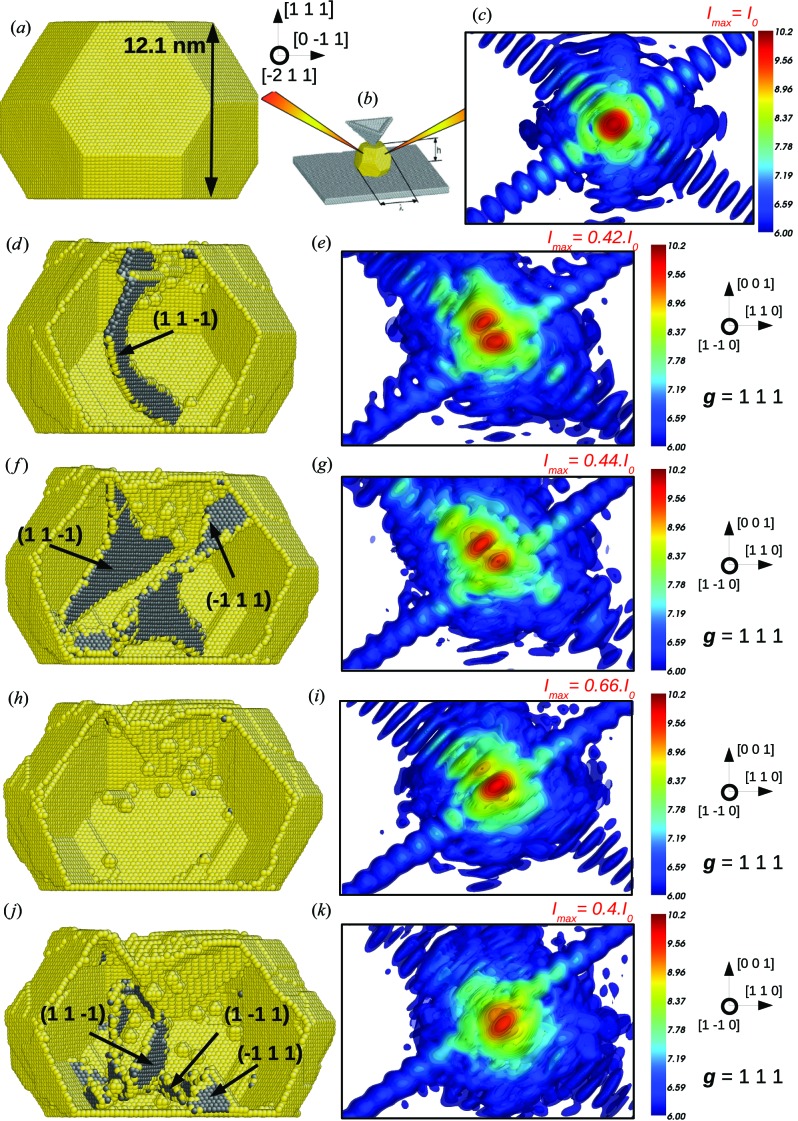
(*a*) Simulation of the indentation of a 12.1 nm high gold nanoparticle by a cube-corner indenter. (*b*), (*c*) Atomistic configuration at the initial state and corresponding CXD pattern (see text for more details) The dislocations are shown in grey. (*d*), (*e*) Gold nanoparticle after 650 000 indentation steps (*t* = 3.25 ns) and calculated CXD pattern. (*f*), (*g*) Gold nanoparticle after 850 000 indentation steps (*t* = 4.25 ns) and calculated CXD pattern. (*h*), (*i*) Gold nanoparticle at *t* = 5 ns and corresponding CXD pattern. (*j*), (*k*) Gold nanoparticle at the final stages of indentation (*t* = 6 ns) and corresponding CXD pattern. The selected area of the reciprocal space is kept to the same value in all figures and is equal to 0.08 × 0.12 Å^−1^.

The Winterbottom construction (Winterbottom, 1967 ▶) is employed, considering the surface energies of the Au potential and the interface energy to initialize the particle configuration (see Fig. 11 ▶ *b*). The indenter in the simulation is lowered at a constant velocity and the integration step is 5 fs. To avoid the complexity of the lattice mismatch between the particle and indenter/substrate, the indenter and substrate are assumed to be much harder than the nanocrystal and are frozen into their perfect crystal locations (Mordehai, Kazakevich *et al.*, 2011 ▶). The effect of the residual strain induced by the substrate is thus not taken into account in this model.

Figs. 11 ▶(*a*) and 11(*c*) show the gold particle in its initial state and the corresponding CXD pattern around the Bragg position **g** = 111, parallel to its upper facet. These are realistic diffraction conditions. Given the smaller size of the particle compared to the reference crystallite (12.1 nm *versus* 30 nm), the calculation of the three-dimensional CXD pattern is done on a larger volume of the reciprocal space: 0.8 × 0.8 × 1.2 Å^−1^. Additionally, the dynamic range is kept to 4.15 decades, but the maximum of intensity is decreased by a factor of 100 (ten times fewer atoms in the particle). Since the crystal is still in its pristine state, the diffraction pattern looks very clean, with a maximum intensity at Bragg positions and rather intense fringes along [111] due to the relatively large size of its (111) facet.

Fig. 11 ▶(*d*) illustrates the atomistic configuration and its corresponding CXD pattern after 650 000 steps of indentation (*t* = 3.25 ns). At this stage of the indentation process, nucleation and glide of multiple dislocations have already occurred, leaving short slip steps on the {111} and {100} facets. A dislocation half-loop with Burgers vector of type **b** = 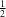
〈110〉 dissociated into partials in one of its {111} slip planes can be seen at the centre of the volume. When looking at the CXD pattern, this defect induces a strong and characteristic signature with intense fringes along [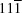] due to the stacking fault, and a splitting related to the phase jump induced by the dislocation half-loop. One can notice that the period of the defect fringes is approximately twice the period of the facet fringes. As stated in previous sections, this is a good indication of the defect location at the centre of the volume. Additionally, since the upper (111) facet is compressed, the period of the fringes along this direction slightly increases.

After 850 000 steps (*t* = 4.25 ns) (Fig. 11 ▶ *f*), the dislocations have left multiple slip steps on the crystal facets, and multiple dislocation half-loops are found in the crystal. The largest loop is dissociated into partials in the (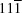) and (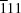) planes, with a Burgers vector along the intersection between these two planes, *i.e.* 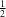
[101]. Correspondingly, the CXD pattern displays intense fringes along the [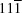] (Fig. 11 ▶ *g*) and [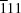] (not shown) directions. The period of the fringes along [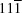] roughly equals four times that of the facet fringes. One can guess that this is due to the decomposition of two dislocations in the (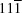) slip plane. Similarly to the previous step, we can observe the Bragg peak splitting into two spots, probably because of the phase jump induced by the main dislocation half-loop.

After deeper indentation, around 106 steps (*t* = 5 ns), no more dislocations can be found remaining in the crystallite (Fig. 11 ▶ *h*). Consequently, the calculated CXD pattern displays a single and clean spot at the Bragg position, and stacking fault fringes along [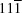] and [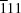] have completely vanished. One can notice that the period of the fringes along [111] increased since the crystal underwent further compression.

At the final stages of the simulated indentation process (*t* = 6 ns), the crystal hosts multiple dislocation loops which decompose into partials in three out of the four available {111} slip planes (Fig. 11 ▶ *j*). The diffraction pattern becomes very difficult to interpret owing to the interplay of multiple defects, and the characteristic signatures such as a splitting or intense fringes along one of the 〈111〉 directions cannot be identified. At this stage, the diffraction pattern is well ‘speckled’ and a statistical interpretation could relay the identification of individual defects as suggested by Favre-Nicolin *et al.* (2010 ▶) and Jacques *et al.* (2013 ▶).

**Figure 12:**
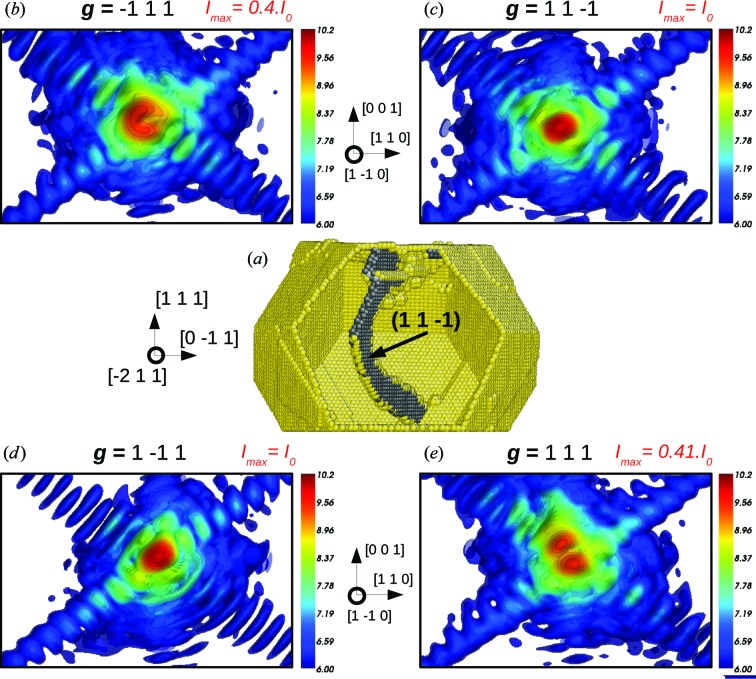
(*a*) Gold nanoparticle after 650 000 indentation steps. A dislocation half-loop with **b** of type 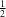
〈110〉 can be observed. (*b*)–(*e*) Calculated CXD patterns for four different 111-type diffraction vectors. The selected area of the reciprocal space is kept to the same value in all figures and is equal to 0.08 × 0.12 Å^−1^.

We now come back to an earlier stage of the indentation (*t* = 3.25 ns), when a single dislocation half-loop can be found in the particle (Fig. 12 ▶). Our goal is to determine the Burgers vector of this loop using the extinction conditions detailed in previous sections. Since this dislocation half-loop is a mixed dislocation, there are no conditions where **g**·**u** is exactly zero everywhere (Hirth & Lothe, 1968 ▶). However, one can assume that the condition **g**·**b** is sufficient to hide most of the dislocation signature in the dislocation pattern. The Burgers vector of the dislocation half-loop is of 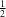
〈110〉 type. Consequently, two of the 〈111〉 diffraction vectors must be perpendicular to **b**. When looking at the calculated CXD patterns for four of the eight 111-type diffraction vectors, one can notice that the signature of the defect is only visible for **g** = 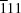 and **g** = 111, whereas no signature can be found for **g** = 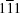 and **g** = 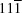 (see Fig. 12 ▶). Both diffraction vectors fulfil the extinction criterion and there is only one possible Burgers vector perpendicular to these two directions: **b** = 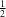
[011]. This demonstrates the possibility to identify both the Burgers vector and the slip plane of a dislocation by the appropriate selection of two, or at most three, diffraction vectors. More generally, this study proves that the technique is adapted to the interpretation of CXD patterns from realistic structures. On the other hand, as shown by the atomistic configuration from the late stages of indentation, the interpretation of CXD patterns from complex structures with multiple defects remains highly challenging owing to the interplay between multiple defects on the corresponding CXD pattern.

## 5. Discussion   

The results above show that all typical defects of f.c.c. crystals induce strong distortions of the CXD patterns at most Bragg reflections. This holds both for dislocations, which induce a long-distance strain field, and for stacking faults, which are nearly strain-free defects. The case of the stacking fault illustrates that coherent X-ray diffraction is, properly speaking, sensitive to the atomic displacement field and not just the elastic strain: in this case it extends over a semi-infinite volume, hence the localized signature at the Bragg peaks. Even better, each defect has a characteristic signature on particular Bragg peaks, such that it can in principle be unambiguously identified from the measurement of one or several reflections. For instance, the characteristic CXD pattern of a perfect screw dislocation at a Bragg reflection not perpendicular to the Burgers vector leaves no ambiguity on the nature of the defect and its Burgers vector. Similarly, characteristic fringes at reflections with *h* ± *k* ± *l* ≠ 3*n* indicate the presence of a stacking fault and reveal its orientation. While these two cases are quite straightforward, the identification can be much more delicate for defects that display complex diffraction patterns such as Frank or prismatic loops. For the latter it appears clearly that several reflections are needed in order to guess what kind of defect the system hosts. For instance, a relaxed Frank loop can be efficiently identified by using two reflections parallel to a partial stair rod and a partial Shockley. Similarly to what has been observed from the elastic diffuse scattering of dislocation loops, the interstitial or vacancy character of a Frank loop (or the intrinsic or extrinsic character of a stacking fault) can also be identified using coherent X-ray diffraction.

For both screw and edge dislocations, the technique can also be used to unambiguously evidence the dissociation into Shockley partials with two very clear and identifiable effects: elongation and splitting of the Bragg peak along **b** and doubling of the fringe period in the direction perpendicular to the dissociation plane (§§3.1 and 3.2). The dissociation of the dislocation is best shown using reflections of high indices, and preferably perpendicular to the Burgers vectors. One can infer that, more generally, such measurement is sensitive to the core structure of the dislocation, since it influences the spatial shift between two sub-volumes of the crystal.

Moreover, even reflections that do not show any distortion can be very useful in establishing the characteristic features of a crystal defect. A wise use of invisibility criteria allows the determination of a dislocation Burgers vector and dissociation plane using only a couple of well chosen reflections. This holds in principle for any kind of single defects that can be encountered in f.c.c. materials.

On the basis of these results, we propose an experimental strategy to identify and characterize a single defect in an f.c.c. crystal.

The first step would be to measure several 111-type reflections in order to distinguish between dislocations and stacking faults/Frank loops: if the defect is a stacking fault or a Frank loop, the defect signature should vanish for only one pair (**g**, −**g**) of these reflections and be visible for every other 111 reflection, whereas the signature will be invisible for two pairs (**g**, −**g**) if the defect is a dislocation. In the first case, a Frank loop is easily distinguished from the stacking faults by the strong distortion at the Bragg position. In the case of dislocations, the Burgers vector can be determined by identifying the two pairs (**g**, −**g**) out of four for which the signature is visible. Until this stage the character of the dislocation does not matter. Once the Burgers vector is established, the use of a reflection **g**|| **b** will allow one to determine the character of the dislocation. A prismatic loop is identified by simultaneous evidence for edge and screw dislocations.

Following this procedure it is in principle possible to determine all the characteristics of a given single defect: for a dislocation, its type, its Burgers vector, its dissociation plane, its dissociation length and an estimate of its position; for a stacking fault, the faulted plane, its extrinsic or intrinsic character (vacancy or interstitial type in the case of a Frank dislocation loop), and a rough estimate of its position.

Regarding experimental matters, it turns out that a high dynamical range is not needed during measurements. In the cases presented here, a single decade of intensity is enough to show a distortion or a splitting of the Bragg peak, and two decades suffice to show a modification of fringes due to a stacking fault. The counting time can thus be significantly reduced, making easier the live monitoring of deformation mechanisms (in such a case, however, the choice of the Bragg reflection for live monitoring implies that some defects remain invisible). The direct analysis of reciprocal space is thus complementary to real-space reconstruction, which requires longer counting times.

An important concern regarding the experimental setup is our ability to resolve the features induced by defects during coherent X-ray diffraction experiments. In fact, the fundamental size of the finest diffraction features on CXD patterns is determined by the size of the diffracting volume (*i.e*. the sample or the beam size, depending on which is smaller). If the experimental setup allows us to sample the reciprocal space with a step size small enough to resolve the fringes induced by the finite size of a perfect crystal, it is also able to resolve any kind of defect signature in a faulted crystal of the same size, independently of the nature and the number of defects. For instance, the splitting distance (*i.e.* the distance between the two maxima of intensity of the split Bragg peak) induced by a dislocation is of the same order of magnitude as the fringe period is related to the crystal size.

Additionally, direct analysis of reciprocal space relies on the comparison between simulation and experimental data. Even if valuable information can be already extracted from the two-dimensional cut of the detector plane, this approach implies that, most of the time, we must record the full three-dimensional CXD pattern in order to produce the needed two-dimensional cuts of reciprocal space. For typical CXD experiments in Bragg geometry with a crystal whose size is around 300 nm (Beutier *et al.*, 2012 ▶; Watari *et al.*, 2011 ▶), the acquisition of a three-dimensional CXD pattern that fulfils the oversampling conditions in the three directions of the space requires that reciprocal space be probed with an extent of approximately ±0.5° and steps of 0.01° (100 points in total). To achieve a dynamical range between four and five decades of intensity, the usual exposure time lies between 2 and 5 s for each point of the rocking curve, and between 200 and 500 s for the acquisition a full three-dimensional CXD pattern.

For a direct analysis of CXD patterns we suggested that a single decade of intensity is sufficient to show a distortion or a splitting of the Bragg peak, while two decades are needed for the modification of fringes due to a stacking fault. The acquisition time can thus be reduced at least by a factor of 50 (0.1 s or even less per point). It would thus only need 4–10 s to perform the acquisition of a full three-dimensional CXD pattern. With only one decade of intensity, the three-dimensional reconstruction of the experimental data is not likely to provide a complete and accurate picture of the strain and defect distributions, while the analysis of the reciprocal space pattern can already provide some information on the latter. To obtain the same kind of dynamical range for a 30 nm crystal (size comparable to the molecular statics simulations), the acquisition time has to be multiplied by 1000. However, for a 300 nm crystal a 0.1 s acquisition time provides almost three decades of intensity. In principle, the acquisition time can be divided by a factor of two or three if one wants to show the perturbations in the crystal fringes, and even 20–30 to highlight the splitting or distortion of the Bragg peak.

One could also wonder if these calculations, performed on f.c.c. metals, are valid for other crystal structures such as hexagonal or body-centred cubic lattices. In the latter, the dislocation structure, its motion and its relaxation are very different from those of f.c.c. crystals. The calculations performed on dissociated dislocations should in principle not be valid for such crystalline structures. However, it appears reasonable to think that the simulations performed for the perfect dislocations and stacking faults are still correct. These perfect defects are described by simple geometric models; only the Burgers vector may differ in other crystal structures. In the case of stacking faults, it induces a different phase shift, and hence a different contrast, but modulated streaks are still expected, provided the planar geometry of the stacking fault is stable. Extinction conditions different from *h* + *k* + *l* = 3*n* will apply. Several studies on materials with the wurtzite and the zinc-blende structures (Chamard *et al.*, 2008 ▶; Favre-Nicolin *et al.*, 2010 ▶; Jacques *et al.*, 2013 ▶) have shown that the phase jump induced by stacking faults in these crystal structures is the same as in f.c.c. structures (±2π/3 depending on the number of faulted planes and the *hkl* indices of the reflection). In the case of the perfect edge and screw dislocations, the displacement fields scale with the Burgers vector, such that the contrast of characteristic features may be different.

If this study establishes the efficiency of CXD to probe single defects, it does not address the case of multiple defects, which can be encountered in various experimental samples. Very few studies have been carried out so far on multiple defects, and they only focus on the case of stacking faults. In such complex systems, as pointed out in §§3.3 and 4, a statistical approach can be used to get relevant information about dislocation density and distribution (Jacques *et al.*, 2013 ▶) or about the stacking fault sequence (Favre-Nicolin *et al.*, 2010 ▶). Alternatively, a wise use of the invisibility conditions can provide very useful information about defect content and density.

A further complication is the interaction of the defects with residual strain in the sample, due for instance to the growth process. Here we discarded this complication to focus on the defects, but in many realistic cases that is a crude approximation, and the calculations presented here are, for instance, not suited to the case of interface dislocations. The study of crystallites in an epitaxial relationship with their substrate, resulting in inhomogeneous strain distribution with a significant contribution of the latter on CXD patterns (Diaz *et al.*, 2010 ▶; Beutier *et al.*, 2012 ▶; Mastropietro *et al.*, 2013 ▶), could be a further development. This would allow one to make comparisons with more realistic experimental cases even if disentangling the contributions of interface strain and defects appears quite challenging.

## 6. Conclusions   

We carried out a detailed numerical analysis of the effect of defects in f.c.c. nanocrystals on their CXD patterns in the vicinity of allowed Bragg reflections. Realistic atomic potentials were used to equilibrate the structures. Our analysis demonstrates the unique character of the signature induced by a single defect and the crucial importance of the diffraction conditions, *i.e.* the selection of the diffraction vector. The relaxation of the faulted crystal structure is shown to have a large impact on CXD patterns. From these characteristic signatures, we suggest a procedure based on the measurement of a few reflections to identify a defect and its characteristics when it is known that it is alone in the structure.

We also extended the scope of this study to nanocrystals containing a few defects by analysing the case of a gold nanocrystal undergoing simulated indentation: we demonstrated that the defects generated in the early stages of indentation can in principle be identified by the study of CXD patterns at several chosen reflections. The use of invisibility conditions proves to be particularly efficient on such complex systems.

Such direct analysis of the reciprocal space requires significantly lower counting times than phase retrieval imaging methods and is well suited to the live monitoring of the nucleation of defects (for instance to study deformation mechanisms during *in situ* loading experiments).

## Figures and Tables

**Table 1:** SFE of five f.c.c. metals, from EAM and experiments, and their _s_/*b*_p_ parameter, the corresponding dissociation length in real space, as obtained after 1600 relaxation steps, splitting distance (see text for more details) and maximum intensity in reciprocal space for **g** = 224

	Ag	Cu	Au	Ni	Al
_s_ (mJm^2^): EAM	17.8 (Williams *et al.*, 2006 ▶)	44.7 (Mishin *et al.*, 2001 ▶)	42.6 (Grochola *et al.*, 2005 ▶)	125.2 (Mishin *et al.*, 1999 ▶)	149.3 (Mishin *et al.*, 1999 ▶)
_s_ (mJm^2^): experiments	16 (Hirth Lothe, 1968 ▶)	45 (Westmacott Peck, 1971 ▶)	32 (Jenkins, 1972 ▶)	125 (Balluffi, 1978 ▶)	144 (Carter Ray, 1977 ▶)
_s_/*b*_p_ (10^3^): EAM	3.5	7.4	9.5	11.7	33.5
Average dissociation length ()	85	37	47	29	18
Splitting distance (10^3^ ^1^)	6.67	15.1	12.9	16.5	17.1
Maximum intensity	1.87 10^11^	2.25 10^11^	2.25 10^11^	2.21 10^11^	2.67 10^11^

**Table 2:** Summary of all the most relevant cases that can be encountered during the study of the signature of single defects For each case the maximum intensity calculated on the CXD pattern is compared with the intensity for a defect-free crystal with the same size and shape. In the following **n** is the direction normal to a stacking fault, **t** is the dislocation line direction, and **b** **t** is the direction perpendicular to both the Burgers vector and the dislocation line direction. The best conditions to show the defect are highlighted in italic, while the invisibility conditions are highlighted in bold. N/A: not applicable; SF: stacking fault.

	Screw dislocation		Edge dislocation				
	Unrelaxed	Relaxed	Unrelaxed	Relaxed	Stacking fault	Frank dislocation loop	Prismatic dislocation loop
**g**(**b** **t**) = 0	**Single clean spot **	**Single clean spot **	**Single clean spot **	Single clean spot, fringes along **n**	N/A	**Small drop of intensity in Bragg position**	**Single clean spot, slight distortion in the pattern**
	*I* = *I*_0_	*I* = *I*_0_	*I* = *I*_0_	*I* = 0.78*I*_0_		*I* = 0.85*I*_0_	*I* = *I*_0_

**g** **b** = 0, **g**(**b** **t** ) 0	**Single clean spot**	*Splitting along **b**, fringes along ***n****	Elongation along **b**	*Splitting along **b**, fringes along ***n****	N/A	Single clean spot, slight disturbances	Single clean spot, drop of intensity in Bragg position
	*I* = *I*_0_	*I* = 0.2*I*_0_	*I* = 0.4*I*_0_	*I* = 0.2*I*_0_		*I* = 0.8*I*_0_	*I* = 0.65*I*_0_

**g**||**b**	*Ring-shaped pattern: ring axis along ***b***; extinction in Bragg position *	Ring-shaped pattern: ring axis along **b**; extinction in Bragg position; maxima of intensity along [001]	*Fringes along ***n***, elongation of Bragg peak along **b***	Fringes along **n**, elongation of Bragg peak along **b** and increased intensity in Bragg position	N/A	*Satellite spot and low intensity in Bragg position *	*Hexagonal-shaped pattern with elongation along ***b***, fringes along ***n***, intensity maximal in Bragg position *
	*I* = 0.14*I*_0_	*I* = 0.18*I*_0_	*I* = 0.18*I*_0_	*I* = 0.33*I*_0_		*I* = 0.3*I*_0_	*I* = 0.5*I*_0_
						*No fringes along **n**: **g** with *h* + *k* + *l* = 3*n**	

**g**||**b**_p_	Ring-shaped pattern: ring axis along **b**; extinction in Bragg position	*Ring-shaped pattern: ring axis along **b**_p_; extinction in Bragg position *	Fringes along **n**, elongation of fringes along **b**, extinction in Bragg position	Fringes and splitting along **n**, elongation of fringes along **b**	N/A	*Distorted ring-shaped pattern for **g**||**b**_SR_ with ring axis along **b**_SR_*	Similar to **g**||**b** with a lower intensity in Bragg position
	*I* = 0.09*I*_0_	*I* = 0.11*I*_0_	*I* = 0.09*I*_0_	*I* = 0.23*I*_0_		*I* = 0.16*I*_0_	*I* = 0.35*I*_0_

**g** with *h* + *k* + *l* = 3*n*	N/A (no SF)	Fringes along **n** disappear	N/A (no SF)	Fringes along **n** disappear	**Single clean spot**	Fringes along **n** disappear	Fringes along **n** disappear
					*I* = *I*_0_		

**g** with *h* + *k* + *l* 3*n*	N/A	Fringes along **n**	N/A	Fringes along **n**	*Intense fringes along **n** and splitting due to the 2/3 phase jump induced by the SF; intensity in Bragg position 25% that of perfect crystal*	N/A	N/A

General **g**	Ring-shaped pattern; ring diameter inversely proportional to crystal size and *hkl* indices	Ring-shaped pattern; distortion and disorientation of the ring depending on the *hkl* indices of the diffraction vector; increase of the maxima of intensity during relaxation	Fringes and splitting along **n** and elongation of Bragg peak and/or of fringes along **b**, depending on the selected diffraction vector	Fringes and splitting along **n** and elongation/ splitting along **b** depending on the selected diffraction vector; increase of the maximum intensity during relaxation	Only two possible cases (see above)	Three main effects: (1) fringes along **n** normal to the SF, (2) ring-shaped pattern along one of the stair-rod partials, and (3) decrease of the intensity of the central spot and appearance of a satellite spot depending on *hkl*	Distorted hexagonal-shaped pattern not oriented along a particular direction and dependent on the *hkl* indices of **g**; maximum intensity lower than that of perfect crystal
